# Dissecting the microbial community structure of internal organs during the early postmortem period in a murine corpse model

**DOI:** 10.1186/s12866-023-02786-0

**Published:** 2023-02-10

**Authors:** Ruina Liu, Kai Zhang, Huan Li, Qinru Sun, Xin Wei, Huiyu Li, Siruo Zhang, Shuanliang Fan, Zhenyuan Wang

**Affiliations:** 1 College of Forensic Medicine, Xi’an Jiaotong University, Xi’an, 710061 China; 2 Xi’an Mental Health Center Hospital, Xi’an, 710061 China; 3 Department of Clinical Laboratory, Shaanxi Provincial People’s Hospital, Shaanxi Xi’an, 710068 People’s Republic of China; 4 Department of Microbiology and Immunology, School of Basic Medical Sciences, Xi’an Jiaotong University, Shaanxi Xi’an, 710061 People’s Republic of China

**Keywords:** Thanatomicrobiome, Body decomposition,16S rRNA, Internal organs

## Abstract

**Background:**

Microorganisms distribute and proliferate both inside and outside the body, which are the main mediators of decomposition after death. However, limited information is available on the postmortem microbiota changes of extraintestinal body sites in the early decomposition stage of mammalian corpses.

**Results:**

This study investigated microbial composition variations among different organs and the relationship between microbial communities and time since death over 1 day of decomposition in male C57BL/6 J mice by 16S rRNA sequencing. During 1 day of decomposition, *Agrobacterium*, *Prevotella*, *Bacillus*, and *Turicibacter* were regarded as time-relevant genera in internal organs at different timepoints. Pathways associated with lipid, amino acid, carbohydrate and terpenoid and polyketide metabolism were significantly enriched at 8 h than that at 0.5 or 4 h. The microbiome compositions and postmortem metabolic pathways differed by time since death, and more importantly, these alterations were organ specific.

**Conclusion:**

The dominant microbes differed by organ, while they tended toward similarity as decomposition progressed. The observed thanatomicrobiome variation by body site provides new knowledge into decomposition ecology and forensic microbiology. Additionally, the microbes detected at 0.5 h in internal organs may inform a new direction for organ transplantation.

**Supplementary Information:**

The online version contains supplementary material available at 10.1186/s12866-023-02786-0.

## Background

Decomposition involves a mosaic ecosystem closely related to biotic factors (such as the corpse, insects, and microbes), and abiotic factors (such as temperature and humidity). This process mobilizes nutrients bound to once-living organisms into the surrounding environment, yielding a group of assembled decomposers to realize nutrient recycling [1]. Exploring the action of these organisms, especially microbes, in the maintenance of intrinsic ecosystems is important. The microbial composition differences seen during decomposition can be directly traced to the status of bacteria present in the earliest stages of decomposition. Microorganisms, important decomposers, are ubiquitous and come from the environment, are scavengers, or were part of the existing microflora of once-living organisms. Briefly after the death of an organism, microbes begin to proliferate, transmigrate and produce specialized proteins that digest host tissues [2]. Moreover, volatile organic compounds associated with the process of bacterial metabolism recruit (or repel) insects [3], which, if given the opportunity, can subsequently eliminate any remaining flesh. Thus, understanding the bacterial basis of decomposition is crucial for understanding decomposition ecology. Additionally, the processes and dynamics of these microbes may be applied in forensic science, particularly concerning estimating the postmortem interval (PMI) [4–6].

Recently, several studies have utilized culture-independent, high-throughput sequencing technologies and bioinformatic analysis to determine the postmortem microbial community composition associated with cadaver skin [1, 4, 7, 8], the oral cavity [7–9], and the intestinal tract [1, 4, 7, 9–11]. Many of these studies have mainly focused on microbial succession of cadavers during the advanced decomposition process (more than 1 day after death) [1, 5, 6, 12] rather than the immediate change after death when there is no obvious putrefaction of the corpse. These studies mainly collected samples from body sites that have indigenous microbes, such as the skin, intestine, and oral cavity. There are few reports about postmortem microbial succession in internal organs [13–16], which are presumed to be sterile [13]. It is important to study microorganisms of internal organs associated with corpse decomposition, because the presence/absence and the abundance of certain bacteria may indicate early PMI (EPMI). Compared to a long PMI (LPMI), EPMI estimation is more significant in forensic practice, as it improves the efficiency of case detection [17–19].

Studies have indicated a rapid microbial succession [20] from aerobic bacteria to anaerobic bacteria [21] in the early decomposition of most body sites. In addition to changes in the microbial communities of specific locations within the body, bacteria can transmigrate from one organ to adjacent organs locally after death, or to distant locations via vascular channels or some unknown mechanisms [22]. Burcham et al. found significant increases in the relative abundance of *Clostridium* in the brain, heart, spleen, and liver samples, during the exudation of body fluids during long-term decomposition [2]. Their study also demonstrated that gene transcripts of multiple metabolic pathways were abundant in these organs, and the transcripts associated with the stress response and dormancy increased during the decomposition [2]. Some studies have utilized intranasal inoculation of *Staphylococcus aureus* and *Clostridium perfringens* to investigate their postmortem structure and functional dynamics [17]. In the heart, liver, spleen, kidney, and other parenchymal organs, the detection rate of *S. aureus KUB7* displayed undulating positivity over time. For example, *S. aureus KUB7* was positive in the kidneys at 1 h postmortem. However, there is limited information about the early changes in the postmortem microbial communities in mammalian animals, especially about the microbiome of extraintestinal locations [18, 19]. Thus, systematic studies are needed to investigate postmortem microbial community changes of internal organs in healthy individuals to provide basic data. In the current study, the microbial composition was characterized and compared between different PMIs and different organs (brain, heart, liver, and kidney). In addition, the shift in microbial assemblages in a mouse 1-day decomposition model was described. Understanding the shifts in microbial community composition and functional-associated pathway levels may be beneficial to studies in decomposition ecology, forensic science, and even organ transplantation.

## Results

### Microbial variation in internal organs after death

#### Relative abundance profiles of the microbial community during 1 day of decomposition

120 organ samples, including those from the brain, heart, liver, and kidneys, were collected from 30 mouse remains at 5 timepoints (0.5 h, 4 h, 8 h, 12 h, 24 h) over 1 day of decomposition. In our study, no valid bacterial sequences were detected in the negative controls, indicating that the sequencing data are reliable. To analyze microbial community succession in every organ, the microbial community composition profiles in the four organs were described separately.

**Fig. 1:**
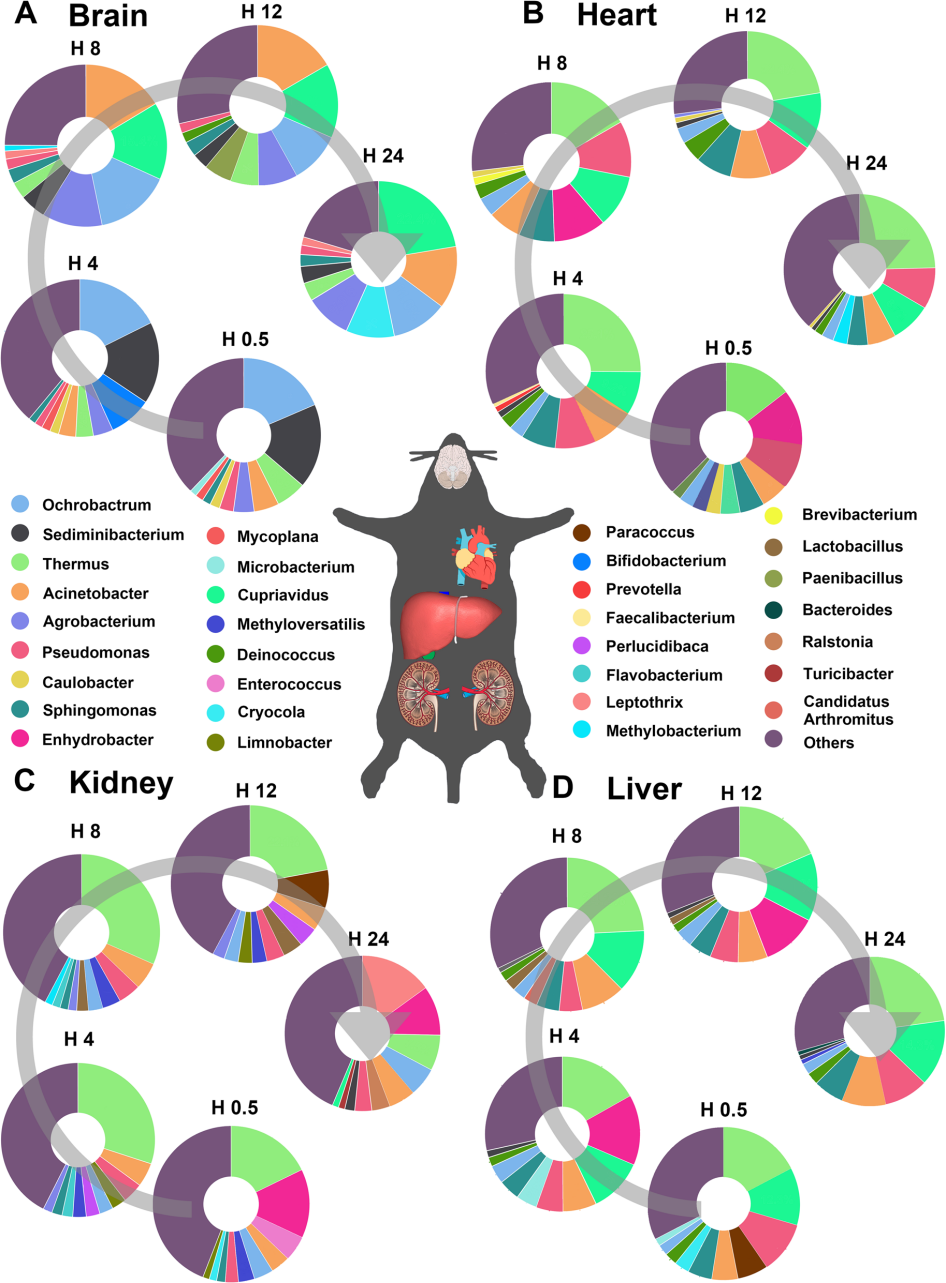
Mean relative abundance of the top 10 bacterial genera identified within each organ during 1 day of decomposition. **A**-**D**: Plots are representative of the brain, heart, kidney, and liver groups, respectively. (Some illustrations were acquired from Wikimedia Commons at http://commons.wikimedia.org/wiki/)

In the brain samples, at the genus level (Fig. 1A and Fig. S1A), *Ochrobactrum* (10.37%—18.54%) and *Sediminibacterium* (3.23%—17.74%) were dominant and showed a similar decreasing relative abundance profile along PMI progression. *Acinetobacter*, *Cupriavidus*, and *Agrobacterium* showed increasing abundance profiles. At the phylum level (Fig. S2A), Proteobacteria (54.06%—87.80%) was the most prevalent in all brain samples. The relative abundance of Bacteroidetes was higher in the H0.5Brain (19.31% ± 0.81%) and H4Brain (19.63% ± 3.27%) samples than in the samples from the other three groups (the *P* values of the six paired comparison groups were all 0.000, *P* < 0.001, LSD t-test). Proteobacteria showed different succession structures during decomposition. In addition, the relative abundance of Thermi illustrated an increase during 12 h of decomposition. At the order level (Fig. S2A), Rhizobiales (21.97%—31.65%) was dominant in all brain samples. The relative abundances of Saprospirales, Caulobacterales, and Thermales decreased, while those of Burkholderiales and Pseudomonadales showed increasing profiles during 1 day of decomposition.

In the heart samples, at the genus level (Fig. 1B and Fig. S1B), *Thermus* (14.52%—25.12%) was more abundant than the other genera in all the heart sample groups. The relative abundances of *Enhydrobacter*, *Caulobacter*, and *Methyloversatilis* gradually decreased during 1 day of decomposition. However, the relative abundance of *Pseudomonas* increased to 11.38% at 8 h after death. The relative abundances of *Sphingomonas* and *Cupriavidus* increased to peak values of 7.93% and 12.58%, respectively, at 12 h after death. At the phylum level (Fig. S2B), Proteobacteria (47.49%—67.28%) and Thermi (16.70%—27.90%) were dominant in the early postmortem heart samples. The relative abundance of Firmicutes gradually increased during 1 day of decomposition, while that of Actinobacteria decreased. At the order level (Fig. S2B), Pseudomonadales (15.21%—27.57%), Thermales (14.52%—25.12%), and Burkholderiales (15.41%—20.26%) were dominant in all heart samples. The relative abundance of Sphingomonadales increased to a peak value of 8.96% at 12 h after death. Rhizobiales showed a gradual increasing abundance profile during 1 day of decomposition. Furthermore, the relative abundance of Deinococcales increased to 4.72% at 12 h after death. However, the relative abundance of Rhodocyclales, Rhodospirillales, and Caulobacterales decreased during 1 day of decomposition.

In the liver samples, at the genus level (Fig. 1C and Fig. S1C), *Thermus* (16.83%—24.22%) and *Cupriavidus* (11.40%—14.35) were dominant in all the postmortem liver sample groups. The relative abundance of *Microbacterium* gradually decreased to zero percent at 24 h after death. In contrast, the relative abundances of *Acinetobacter*, *Cupriavidus*, and *Pseudomonas* gradually increased during decomposition. The genera *Paracoccus* and *Cryocola* were detected only half an hour after death. At the phylum level (Fig. S2C), Proteobacteria (48.29%—62.36%) and Thermi (18.79%—25.39%) were dominant in all the liver sample groups. Actinobacteria, Firmicutes, Bacteroidetes, and Cyanobacteria showed relative abundances of more than 1% in all the liver sample groups. Among these phyla, Actinobacteria gradually decreased in relative abundance during 1 day of decomposition. At the order level (Fig. S2C), Burkholderiales (15.25%—20.72%), Pseudomonadales (14.59%—27.66%), and Thermales (16.83%—24.22%) were dominant in all the liver sample groups. The relative abundance of Clostridiales gradually increased during 1 day of decomposition, while that of Actinomycetales decreased during decomposition. Rhodobacterales immediately decreased in relative abundance during 4-h decomposition.

In the kidney samples, at the genus level (Fig. 1D and Fig. S1D), *Thermus* (7.40%—31.59%) was dominant. The relative abundances of *Acinetobacter* and *Pseudomonas* increased during 8 h of decomposition. The relative abundance of *Methyloversatilis* decreased during 1 day of decomposition. At the phylum level (Fig. S2D), Proteobacteria (41.37%—60.51%), Thermi (8.00%—32.14%), and Firmicutes (7.35%—10.79%) were dominant in all the postmortem kidney sample groups. The relative abundances of Fusobacteria and Cyanobacteria gradually decreased during 1 day of decomposition, while those of Proteobacteria and Actinobacteria gradually increased during decomposition. At the order level (Fig. S2D), Pseudomonadales (11.94%—22.23%) and Thermales (7.40%—31.59%) were dominant orders in all the kidney sample groups. During 1 day of decomposition, the relative abundances of Streptophyta, Clostridiales, and Rhodocyclales gradually decreased. However, the relative abundances of Burkholderiales, Rhizobiales, Bacteroidales, and Actinomycetales gradually increased during this decomposition period.

#### Comparison of alpha and beta diversity at different time points

**Fig. 2:**
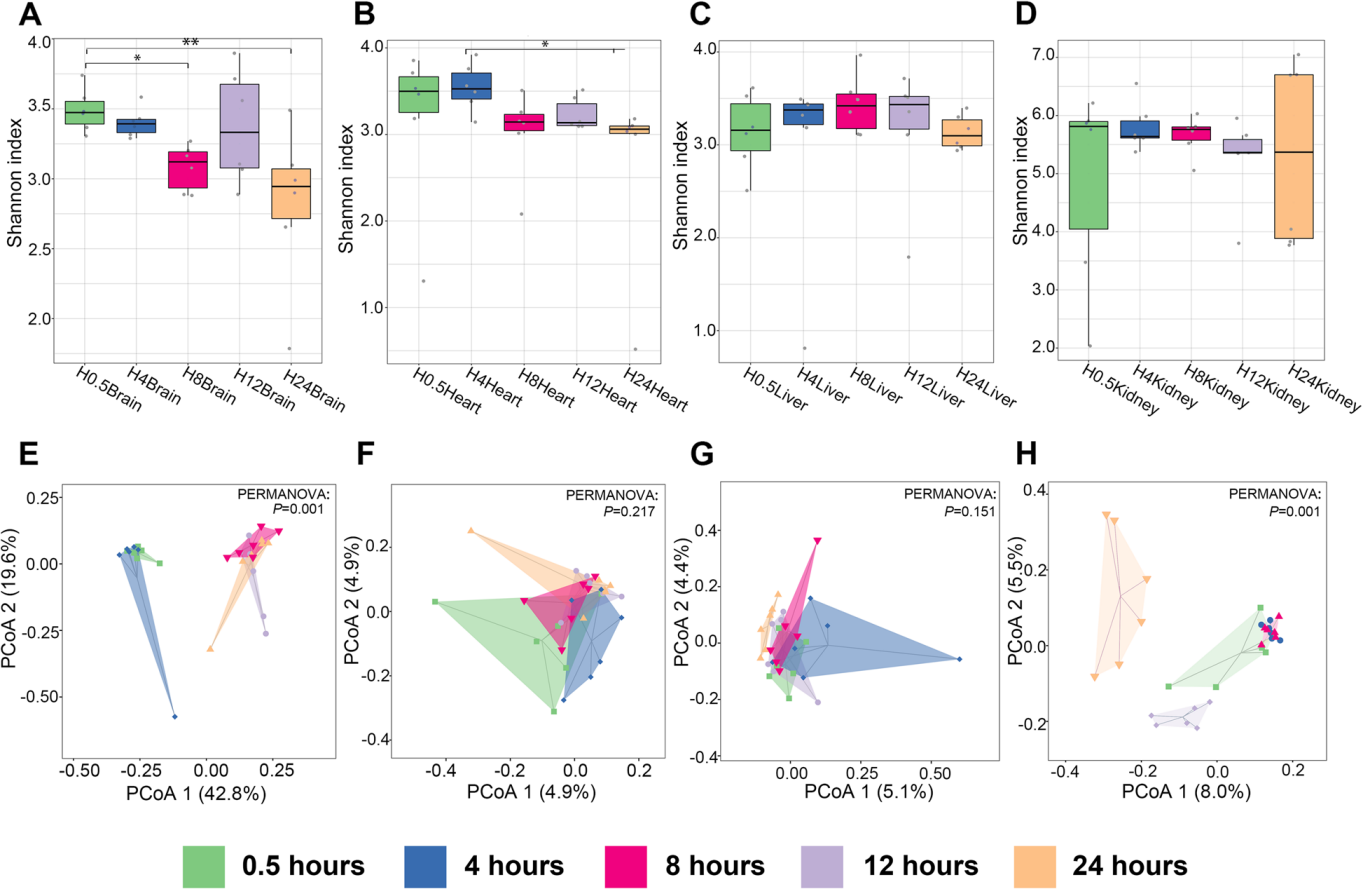
Alpha and beta diversity changed during 1-day decomposition. **A**-**D**: Alpha diversity within subjects at different PMIs in different internal organs (brain, heart, liver, kidney) as measured using the Shannon index. *P value < 0.05, **P value < 0.01, ***P value < 0.001, 6 samples per group. **E**–**H**: PCoA plots based on weighted UniFrac distances of different postmortem groups in brain, heart, liver and kidney samples

Alpha diversity was measured by the Shannon index, Pielou’s evenness, Good’s coverage index, observed species index, Faith’s PD (Faith’s phylogenetic diversity) index, and Chao1 index in this work. Samples in the H8Brain and H24Brain groups had significantly lower alpha diversity (measured as Shannon diversity, H8 v.s. H0.5, *P* = 0.035; H24 v.s. H0.5, *P* = 0.003 (Fig. 2A), Pielou’s evenness, H8 v.s. H0.5, *P* = 0.009; H24 v.s. H0.5, *P* = 0.046 (Fig. S3A), and Good’ s coverage index H8 v.s. H0.5, *P* = 0.028; H24 v.s. H0.5, *P* = 0.035 (Fig. S3C)) than those in the H0.5Brain group (*P* < 0.05, KW-test). Faith’s PD index, indicating the phylogenetic distances of OTUs in a sample, had a significantly lower value in the H0.5Brain group than in the H8Brain (*P* = 0.0015), H12Brain (*P* = 0.014), and H24Brain (*P* = 0.0024) groups (*P* < 0.05, LSD t-test, Fig. S3B). Faith’s PD index was significantly lower in the H24Liver group than in the H4Liver (*P* = 0.009) and H8Liver (*P* = 0.003) groups (*P* < 0.01, Dunnett T3 test, Fig. S3D). According to a comparison of alpha diversity in the kidney groups, the observed species index demonstrated a significantly lower value in the H12Kidney group than in the H4Kidney (*P* = 0.01) and H8Kidney (*P* < 0.0001) groups (*P* < 0.01, Dunnett T3 test, Fig. S3E). The samples in the H12Kidney group showed a significantly lower value of Chao 1 index than those in the H4Kidney (*P* = 0.01) and H8Kidney (*P* < 0.0001) groups (*P* < 0.01, Dunnett T3 test, Fig. S3F).

To visualize the similarities and dissimilarities in community composition between samples, as a measure of beta diversity, PCoA (principal coordinate analysis) plots were calculated based on the weighted UniFrac dissimilarity index. Overall, PCoA of brain samples revealed two distinct clusters (the PCoA1 and PCoA2 explained 42.8% and 19.6% of the variation): one including samples of the H0.5Brain and H4Brain groups and another including samples from the H8Brain, H12Brain, and H24Brain groups (Fig. 2E). PCoA of kidney samples showed three clusters: a cluster of samples from the H0.5Kidney, H4Kidney, and H8Kidney groups; a cluster of samples from the H12Kidney group; and a cluster of samples from the H24Kidney group (Fig. 2H). Liver and heart samples did not show significant clusters according to beta diversity analysis (Fig. 2).

**Fig. 3:**
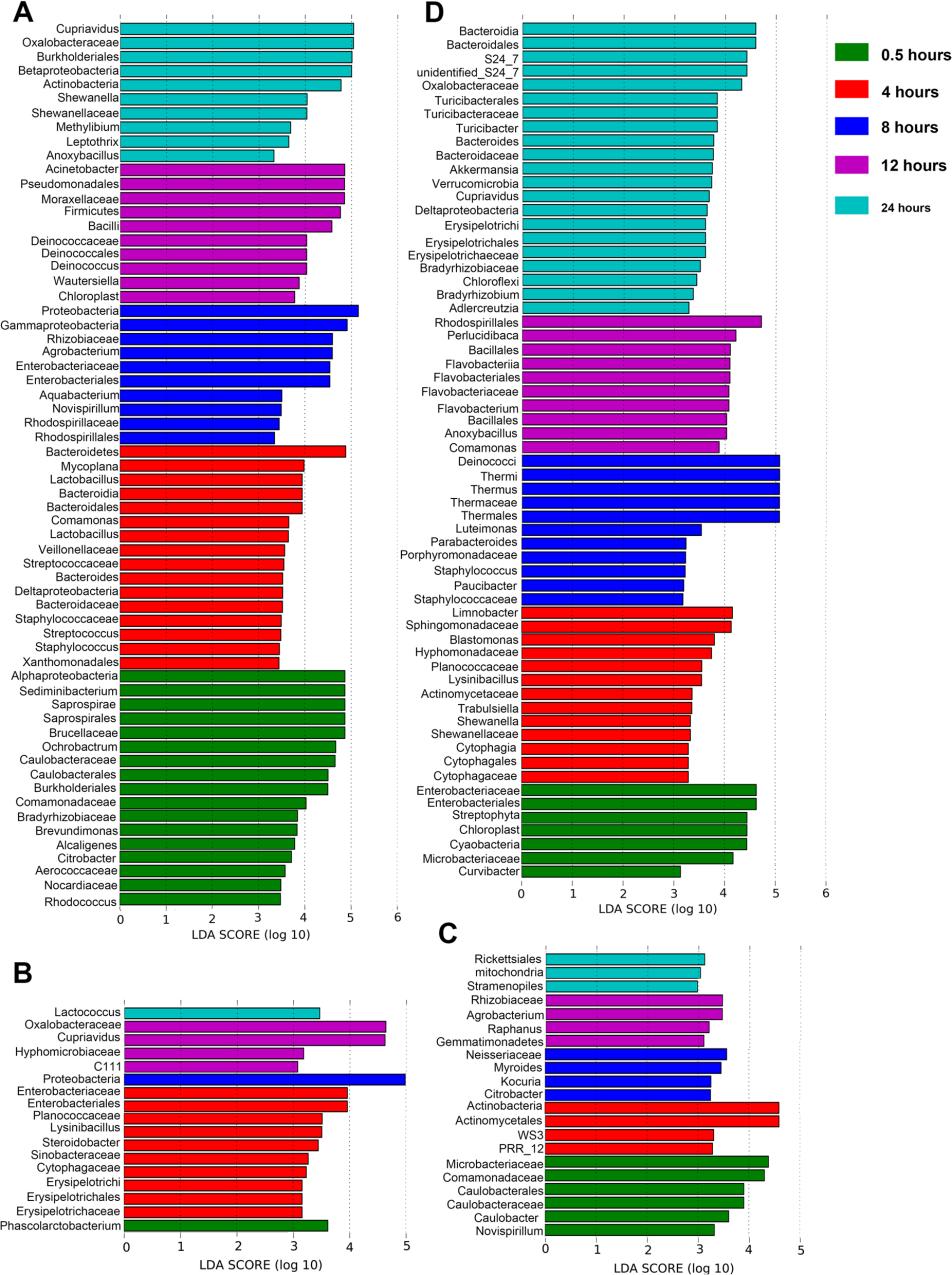
LEfSe rank plots of different organs among different decomposition stages. **A**-**D**: Plots are representative of the brain, heart, liver, and kidney groups, respectively

To identify the taxonomic bacteria with significantly different abundances among different PMIs for internal organs, the linear discriminant analysis (LDA) effect size (LEfSe) method was used for biomarker analysis (Fig. 3) [23]. As shown in Fig. 3A, *Sediminibacterium* and *Ochrobactrum* were representative genera (higher relative abundance) in the H0.5Brain group compared to other brain groups. *Lactobacillales* and *Mycoplana* were significant microbes in the H4Brain group. *Agrobacterium* was the significant genus in the H8Brain group. *Deinococcus* and *Acinetobacter* were representative microbes in the H12Brain group. *Cupriavidus* and *Cryocola* were representative microbes in the H24Brain group.

When the taxonomic composition was compared within different decomposition stages in the heart (Fig. 3B), *Phascolarctobacterium*, *Xanthomonadaceae*, *Sphingobium*, and *Ellin6529* were significant microbes in the H0.5Heart group; *Enterobacteriaceae*, *Lysinibacillus*, and *Steroidobacter* were detected as representatives in the H4Heart group; Proteobacteria was significant in the H8Heart group; *Cupriavidus* was regarded as a significant microbe in the H12Heart group; *Lactococcus* showed significance in the H24Heart group. Regarding the liver groups, Microbacteriaceae, Comamonadaceae, and *Caulobacter* were significant microbes in the H0.5Liver group (Fig. 3C); Actinomycetales, PRR_12, and Solibacterales were representative microbes in the H4Liver group; *Myroides*, *Kocuria*, *Citrobacter*, and Streptomycetaceae were significant microbes in the H8Liver group; Gemmatimonadetes and *Raphanus* were representatives in the H12Liver group; *mitochondria* were regarded as significant microbes in the H24Liver group. For the kidney groups (Fig. 3D), Enterobacteriaceae and Streptophyta were significant microbes in the H0.5Kidney group; *Limnobacter*, Sphingomonadaceae, Clostridiales, Sphingomonadaceae, Hyphomonadaceae, and Planococcaceae were significant in the H4Kidney group; *Thermus*, Bacteriovoracaceae, and *Luteimonas* were significant in the H8Kidney group; Rhodobacterales, *Perlucidibaca*, Bacillales, *Flavobacterium*, *Anoxybacillus*, *Comamonas*, and Rhodospirillaceae were significant in the H12Kidney group; and *S24_7*, Oxalobacteraceae, *Turicibacter*, *Bacteroides*, *Akkermansia*, *Cupriavidus*, and Deltaproteobacteria were significant microbes in the H24Kidney group.

#### Co-occurrence network analysis of postmortem microbial ecology

A co-occurrence network of each group was constructed to explore microbial interactions during decomposition. The number of positive edges in brain groups was more than that of negative edges. With PMI processed, the number of negative edges in the brain increased from 5 to 19 (Fig. S4). In heart groups, the dominant genus *Thermus* had positive correction with *Pseudomonas*, *Ralstonia*, and *Acinetobacter*. *Sphingomonas* in the H0.5Heart group had 2 positive edges with *Brevundimonas* and *Caulobacter*, while 7 negative edges with taxa from *Ochrobactrum*, *Sediminibacterium,* and *Ralstonia* (Fig. S5A). *Sphingomonas* in the H4Heart group had 2 positive edges with *Brevundimonas* and *Caulobacter*, while 7 negative edges with taxa from *Pseudomonas* and *Sediminibacterium* (Fig. S5B). *Sphingomonas* in the H8Heart group had 2 positive edges with *Brevundimonas* and *Caulobacter*, while 10 negative edges with taxa from *Pseudomonas*, *Acinetobacter*, *Ochrobactrum*, *unidentified_MLE1 − 12*, and *Sediminibacterium* (Fig. S5C). *Sphingomonas* in the H12Heart group had 2 positive edges with *Brevundimonas* and *Caulobacter*, while 7 negative edges with taxa from *Pseudomonas*, *Ochrobactrum*, *unidentified_MLE1 − 12*, and *Sediminibacterium* (Fig. S5D). *Sphingomonas* in the H24Heart group had 2 positive edges with *Brevundimonas* and *Caulobacter*, while 7 negative edges with taxa from *Ochrobactrum*, *Pseudomonas*, *unclassified_Pseudonocardiaceae*, *unidentified_MLE1 − 12*, and *Sediminibacterium* (Fig. S5E). The microbial community-associated networks in liver groups (Fig. S6) were similar to that in heart groups. *Perlucidibaca* in H0.5, H4, H8, and H12 Kidney groups had 3 positive edges with taxa in *Rhodococcus* and *unidentified_S24 − 7*, while no correction with other taxa in H24Kidney (Fig. S7).

### Differential postmortem microbial community structure and diversity between different organs

**Fig. 4:**
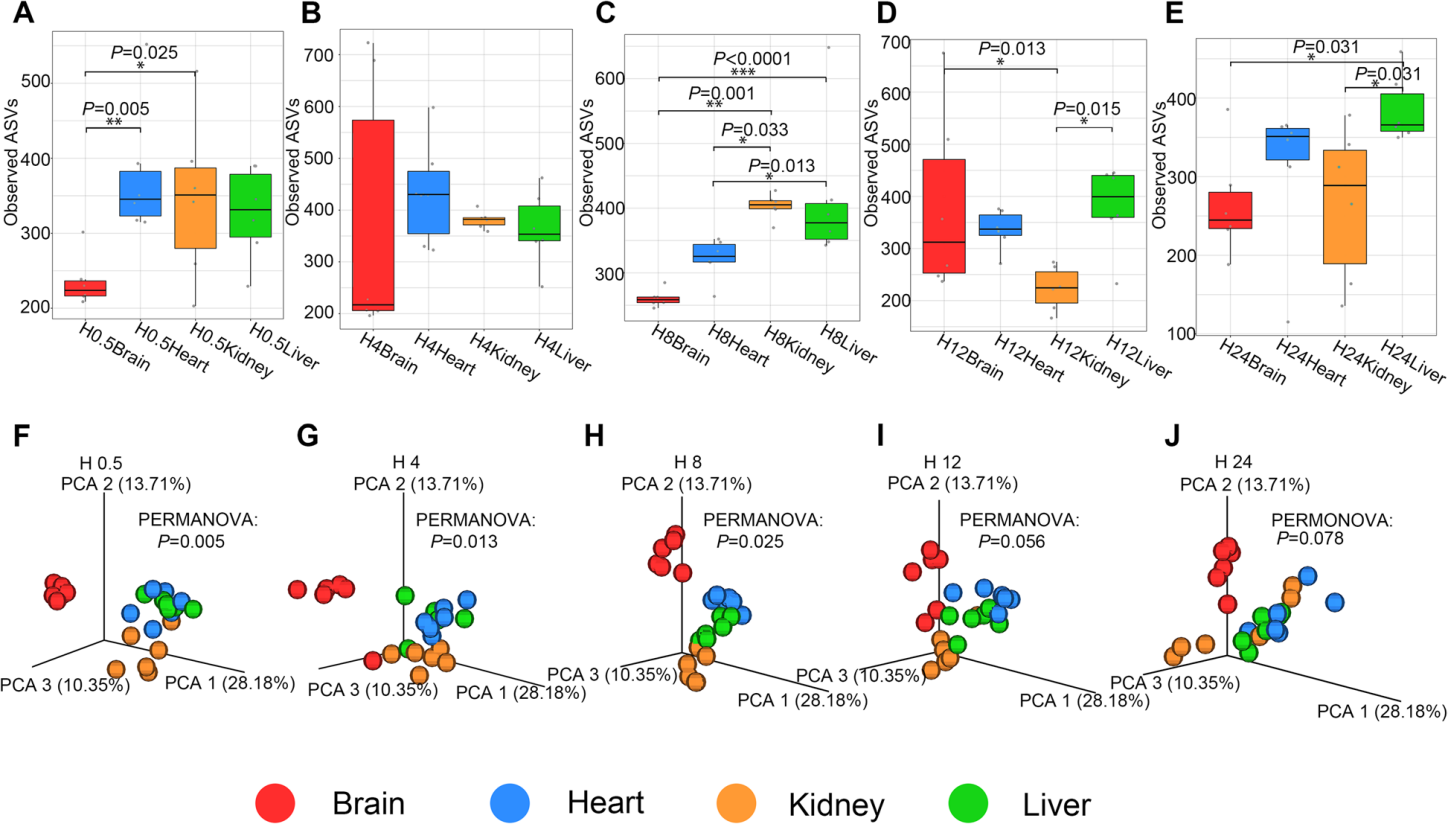
Alpha and beta diversity measures of microbial communities by organ. **A**-**E**: Observed ASV richness by organ. *P < 0.05, **P < 0.01, ***P < 0.001; statistical methods: KW-test with Bonferroni correction or Student’s t-test with Fisher's LSD; sample size: 6 samples per group. H0.5Brain v.s. H0.5Heart, *P* = 0.005; H0.5Brain v.s. H0.5Kidney, *P* = 0.025; H8Brain v.s.H8Kidney, *P* = 0.001; H8Brain v.s. H8Liver, *P* < 0.0001; H8Heart v.s. H8Kidney, *P* = 0.033; H12Brain v.s. H12Kidney, *P* = 0.013; H12Kidney v.s. H12Liver, *P* = 0.015; H24Brain v.s. H24Liver, *P* = 0.031; H24Kidney v.s. H24Liver, *P* = 0.031. (F)-(J): PCoA based on weighted UniFrac measures of microbial dissimilarity by organ

**Fig. 5:**
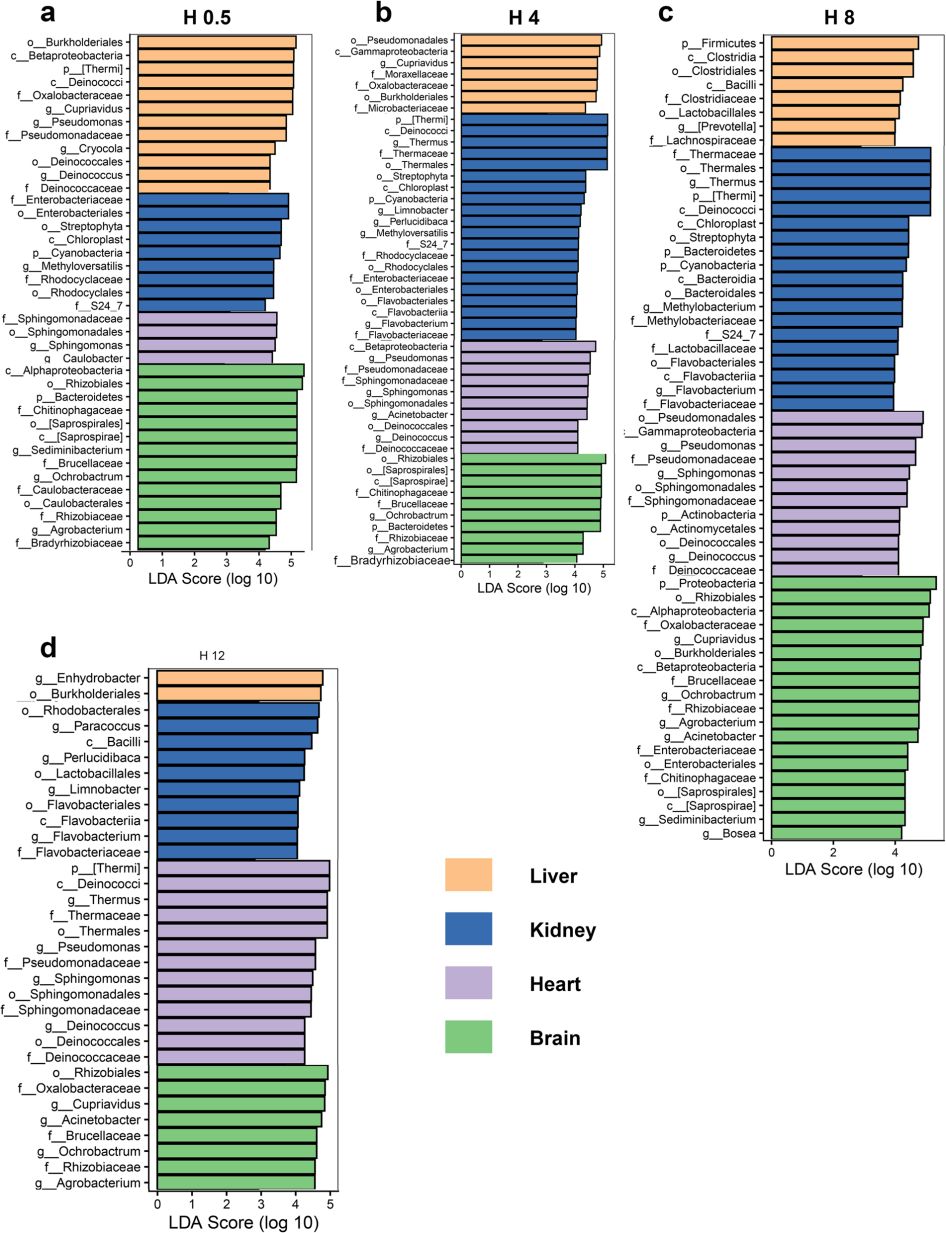
LEfSe analysis of the differential abundance of individual bacterial OTUs between each organ compared to all other organs at each PMI (the LDA threshold score was set as 4.0). **A**-**D**: demonstrated the LEfSe results of 0.5, 4, 8, and 12 h after death, respectively

Alpha diversity was significantly different between the brain and the other organs (heart, kidney, and liver) (*P* < 0.05, Student’s t-test (LSD), KW-test, Fig. 4). The brain groups exhibited lower observed amplicon sequence variant (ASV) richness values than the other organs at half an hour (Fig. 4A, *P*< 0.05), 8 h (Fig. 4C, *P*< 0.05), 12 h (Fig. 4D, *P*< 0.05), and 24 h (Fig. 4E, *P*< 0.05) after death. The exact *P* value of each statistical analysis was shown in Fig. 4. Kidney samples showed the lowest observed ASV richness values among the other organ groups at 12 h after death (Fig. 4D, *P*< 0.05). Beta diversity based on weighted UniFrac distance showed obvious clustering at the early decomposition stage according to the organ, while the distinction decreased with the decomposition process (Fig. 4). In general, the brain samples were dominated by the bacterial genera *Acinetobacter*, *Cupriavidus*, *Ochrobactrum*, and Sediminibacterium, while the other organs were dominated by *Thermus*, *Enhydrobacter,* and *Pseudomonas* (Fig. 1). LEfSe analysis results showed representative microbes among the different sample sites during decomposition at different taxonomic levels (Fig. 5). The number of significant taxa in each organ increased before 8 h and decreased from then on, leading to no significantly differentially abundant taxa in any organ at 24 h after death, with a linear discriminant analysis (LDA) threshold of 4 (Fig. 5). Half an hour after death (Fig. 5A), *Agrobacterium*, *Sediminibacterium*, and *Ochrobactrum* were detected as significantly differentially abundant genera in the H0.5Brain group; Comamonadaceae and *Sphingomonas* were detected in the H0.5Heart group at multiple levels; *Pseudomonas*, *Cupriavidus*, *Deinococcus*, and *Cryocola* were significantly differentially abundant genera in the H0.5Liver group; and *Enterobacteriaceae*, Streptophyta and *Methyloversatilis* were significantly differentially abundant bacteria in the H0.5Kidney group. After 4 h of decomposition (Fig. 5B), Bradyrhizobiaceae and *Agrobacterium* were significantly differentially abundant microbes in the H4Brain group; *Pseudomonas*, *Acinetobacter,* and *Sphingomonas* were detected as representative genera in the H4Heart group. *Thermus*, *Limnobacter*, *Perlucidibaca*, *Methyloversatilis,* and *Flavobacterium* were significantly differentially abundant genera in the H4Kidney group. *Moraxellaceae* and *Cupriavidus* were detected as significantly differentially abundant microbes in the H4Liver group. After 8 h of decomposition (Fig. 5C), *Cupriavidus*, *Ochrobactrum*, *Agrobacterium*, *Sediminibacterium,* and *Acinetobacter* were significantly differentially abundant genera in the H8Brain group; *Sphingomonas*, *Pseudomonas,* and *Deinococcus* were significantly differentially abundant genera in the H8Heart group. In the H8Kidney group, *Thermus* and *Methylobacterium* were significantly differentially abundant genera. After 12 h of decomposition (Fig. 5D), *Acinetobacter*, *Cupriavidus*, *Acinetobacter,* and *Agrobacterium* were detected as significantly differentially abundant genera in the H12Brain group; *Thermus*, *Pseudomonas*, *Sphingomonas,* and *Pseudomonas* were representative genera in the H12Heart group. *Perlucidibaca* and *Flavobacterium* were significantly differentially abundant genera in the H12Kidney group. The genera with the continuous difference in the comparison of four organs were counted based on the LDA effect size of the LEfSe analysis results (Table 1).

**Table 1:** Differential genera associated with different organs during decomposition based on Lefse analysis

Group	Genera	*P* value/ LDA score at hour 0.5	*P* value/ LDA score at hour 4	*P* value/ LDA score at hour 8	*P* value/ LDA score at hour 12	trend^a^
Brain	Ochrobactrum	0.0041/ 4.9100	0.0402/ 4.8934	0.0042/ 4.7879	0.0100/ 4.6123	up
	Agrobacterium	0.0018/ 4.2848	0.0007/ 4.2821	0.0004/ 4.7662	0.0007/ 4.5610	up
	Leptothrix	0.0012/ 3.5893	0.0013/ 3.6662	0.0001/ 3.8906	0.0009/ 3.7786	up
	Aminobacter	0.0032/ 3.5617	0.0354/ 3.6697	0.0041/ 3.6722	0.0124/ 3.4371	up
	Bradyrhizobium	0.0024/ 3.5432	0.0014/ 3.5230	0.0034/ 3.5848	0.0228/ 3.3360	up
	Phyllobacterium	0.0029/ 3.3562	0.0009/ 3.4450	0.0038/ 3.5664	0.0005/ 3.2525	up
Heart	Sphingomonas	0.0033/ 4.2528	0.0022/ 4.4560	0.0003/ 4.4591	0.0006/ 4.5032	up
Kidney	Lysinibacillus	0.0040/ 3.3069	0.0008/ 3.4894	0.0021/ 3.2596	0.0007/ 3.1509	up
	Perlucidibaca	0.0035/ 3.6762	0.0007/ 4.1782	0.0002/ 3.8716	0.0003/ 4.2633	up
	Limnobacter	0.0004/ 3.7829	0.0024/ 4.2050	0.0029/ 3.7611	0.0159/ 4.1156	up

^a^The trend means that the relative abundance of the corresponding genus was increased or decreased compared to other organ group at the same PMI

### Metabolic pathways in postmortem organs during decomposition

**Fig. 6:**
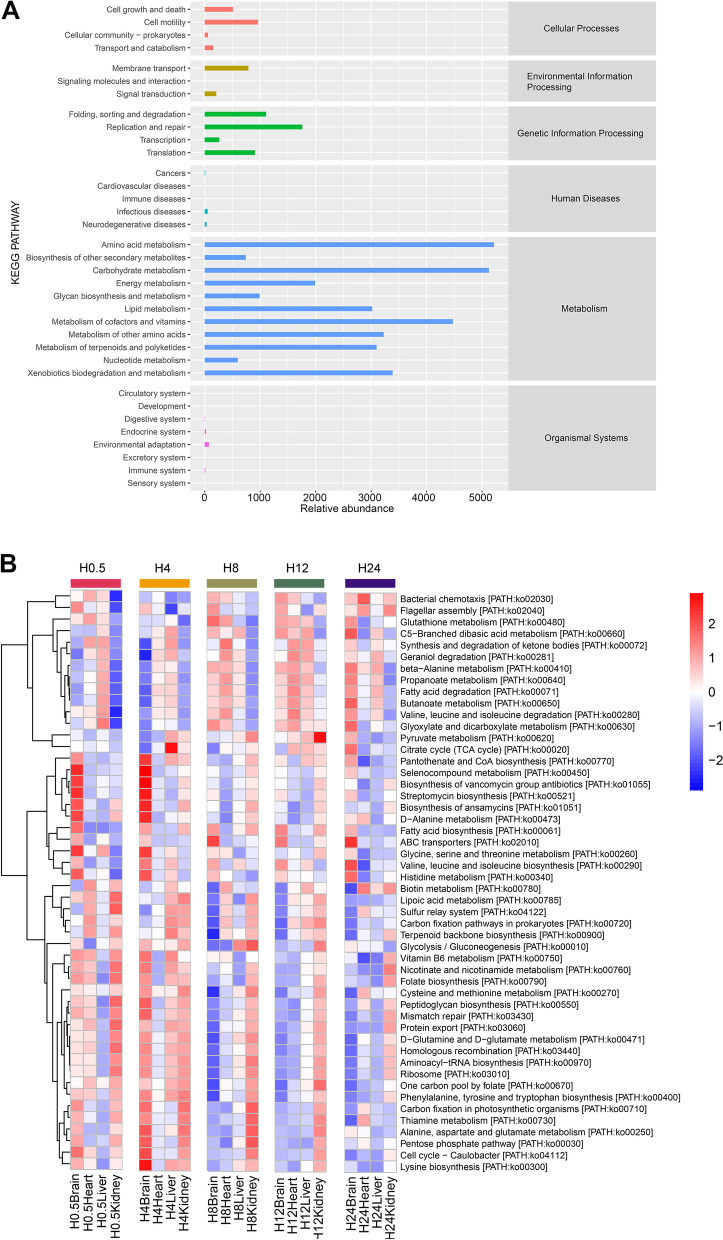
Predictive metabolic potential (from amplicon data) of postmortem internal organs. **A** Distribution of major metabolic KEGG categories across the samples. **B** Heatmap showing the distribution of genes based on level 2 KEGG orthologous gene identification by PICRUSt2 analysis

The metabolic pathways based on the Kyoto Encyclopedia of Genes and Genomes (KEGG) [24] database were used to link microbial genomic information by the Phylogenetic Investigation of Communities by Reconstruction of Unobserved States 2 (PICRUSt2) algorithm [25]with higher-order functions that were significantly altered during 1-day decomposition. To facilitate writing, the organ groups harvested before 4 h after death were called “before 4 h”, while those harvested after 4 h of decomposition were called “after 4 h”. Broad classes of metabolic pathways within the samples are presented in Fig. 6. The enriched pathways of the current study were amino acid metabolism, carbohydrate metabolism, cofactor and vitamin metabolism, xenobiotic biodegradation and metabolism, other amino acid metabolism, terpenoid and polyketide metabolism, lipid metabolism, and energy metabolism, which are summarized in Fig. 6A. The top 5 relative abundance pathways were similar in the four organs. Certain pathways included ketone body synthesis and degradation; ansamycin biosynthesis; valine, leucine, and isoleucine biosynthesis; C5-branched dibasic acid metabolism; and fatty acid biosynthesis. A heatmap shows particular pathways that were differentially abundant according to the organ (Fig. 6B). According to comparisons of different organs at 0.5 h, selenocompound metabolism, histidine metabolism, and several amino acid metabolism pathways were more enriched in the brain than in the other organs. The bacterial chemotaxis and flagellar assembly pathways were lower in kidney samples. After 4 h of decomposition, pantothenate and CoA biosynthesis, selenocompound metabolism, and amino acid metabolism were enriched in the H4Brain group compared with the other organ groups. After 8 h of decomposition, the biotin, lipoic acid metabolism, and carbon fixation in prokaryotes and terpenoid backbone biosynthesis pathways were depleted in brain samples compared to those in samples from the other organs. After 12 h of decomposition, the pyruvate metabolism pathway was enriched in the kidney compared with the other organs. The result of LEfSe analysis showed that metabolic pathways had differential abundance in different PMI points, especially in brain samples (Figs. S8 and S9). Alpha-linolenic acid metabolism had a higher abundance in H0.5Brain than H8Brain, and H24Brain (*P* < 0.01). Arachidonic acid metabolism had a higher abundance in H12Brain than H0.5Brain (*P* < 0.05). Fatty acid elongation in mitochondria had a higher abundance in H4Brain than H24Brain, and H8Brain (*P* < 0.01). Protein digestion and absorption pathway had a higher abundance in H4Brain than H12Brain (*P* < 0.001). Flavonoid biosynthesis and stedroid biosynthesis pathways showed higher abundances in H12Brain than H4Brain (*P* < 0.001).

## Discussion

The common thanatomicrobiome sampling method in previous studies was continuous swabbing in the same bodies. Here, different individuals were sampled each time, rather than repeatedly sampling the same bodies, to prevent exposure of internal organs to the surrounding environment and leading to potential microbiota-related changes. Through 16S rRNA MiSeq sequencing, the temporal dynamics of microbial community composition and predicted gene function within cadavers was observed As posited, the microbial abundance and composition appeared to notably shift along with PMI timepoints in different organs. The differences were most striking in the brain (Fig. 2 and Fig. S3). *Acinetobacter* immediately increased in abundance at hour 8 in brain samples (Fig. S1), which is an aerobic, non fermentative, gram-negative pathogenic bacilli and previously reported in a swine carcass using fluorescence in situ hybridization (FISH) [26]. *Cupriavidus* and *Agrobacterium* were increased after 8 h of decomposition in brain samples. A previous study reported the decomposition ability of *Cupriavidus basilens* in the organic matter since it accelerates organic phosphate decomposition, which could acidify microbial cells and cause the release of orthophosphate from mineral phosphate [27]. At the phylum level, *Bacteroidetes* and *Proteobacteria* showed different succession patterns during decomposition, which could be important information in PMI estimation. The phyla *Proteobacteria* and *Bacteroidetes* are reported to participate in organic matter degradation and carbon turnover [28].

When analyzing the microbial succession of internal organs during decomposition, some interesting findings were observed. *Proteobacteria* and *Cupriavidus* were differentially abundant in the H8Heart and H12Heart groups, which was similarly observed in the brain sample groups. In the heart samples, *Enhydrobacter* and *Caulobacter*, belonging to Alphaproteobacteria, showed a decreasing abundance profile. *Caulobacter* uses energy-dependent proteases to control protein destruction in a highly specific manner [29]. *Methyloversatilis* and *Sphingomonas* are reportedly decomposers that have been isolated from various environments and were also detected in postmortem heart samples in this work [30]. In the liver groups, *Microbacterium*, which gradually disappeared with body degradation and includes aerobiotic bacteria that utilize riboflavin (vitamin B2) as a carbon source [31], was identified and has been reported previously in murine decomposition [32]. *Paracoccus* and *Cryocola* were observed in the H0.5Liver group. *Paracoccus* is a biochemically versatile genus possessing a variety of metabolic pathways through which a wide range of diverse compounds can be degraded. *Cryocola* was found in the co-composting of organic wastes [33]. Comamonadaceae, a family of *Betaproteobacteria,* was also significantly enriched in the H0.5Liver group, meaning that the nutrition of the liver was sufficient at the beginning of death. With oxygen consumption, Actinomycetales, gram-positive and anaerobic bacteria, were dominant in the H4Liver group. This order can be found mostly in soil and decaying organic matter. Rickettsiales was detected as a representative order of the H24Liver group. All bacteria of this order are short coccobacillary organisms, strictly intracellular bacteria, and thus need to survive in an animal host, mainly in arthropods. Rickettsiales may suggest that arthropods are involved in body decomposition 24 h after death. The representative microbes shift from facultative anaerobes (Enterobacteriaceae, Curvibacter) to aerobic bacteria (*Limnobacter*, *Deinococcus*, and Rhodobacterales), and then to obligate anaerobes (*Bacteroides*, *Adlercreutzia*) in kidney samples (Fig. 3D). Enterobacteriaceae, a group of enteric facultative anaerobes, have a fermentative metabolism, which may utilize easily assimilable nutrients after the autolysis process [34]. The reason for the transition from facultative anaerobes to aerobes remains to be further investigated. Several previous studies reported that the organ thanatomicrobiome changed from facultative anaerobes to obligate anaerobes [13, 35]. *Leptothrix was* observed in the H24Kidney group, which can generally be found in environments with sufficient organic matter. It was also reported in closed organs of drowning corpses [36]. Meanwhile, *Lactobacillus reuteri*, one of the most ubiquitous species of mammalian gut microbes, had an increasing abundance profile during 12 h of decomposition in the kidney samples. These results suggested the possibility of gut microbes participating in body decomposition after death.

Microbial alpha diversity showed a slightly fluctuating decrease during 1 day of decomposition, as was observed previously for bacterial alpha diversity in human [1], porcine [8], and murine [5] remains. This study demonstrated that the microbial community evenness of the H0.5Brain group was higher than that of the other brain sample groups according to a higher Pielou’s evenness value. Faith’s PD index comparison among the brain sample groups indicated that the microbial species affinity in postmortem organs decreased with time. Additionally, the decline in Good’s coverage index showed that the proportion of species detected decreased with brain decomposition. The alpha diversity change trends in other organs were not as obvious as those in the brain groups. It was supposed that the incongruence of decay stages among different organs occurred mainly because of their microbial composition at the moment of death. The brain samples formed two clusters in a PCoA plot based on weighted UniFrac distance according to the decomposition stage. In addition, the groups separated in the PCo1 direction explained 42.8% of the variance, which was higher than that for the PCoA of the other organs. These results all illustrated obvious microbial composition succession in postmortem brain samples. Some marked pathways predicted to be associated with organic synthesis and degradation processes, including lipid metabolism, terpenoid, and polyketide metabolism, amino acid metabolism, and carbohydrate metabolism were enriched in our samples (Fig. S5). These findings provide future research directions for the related analysis of microbial communities and metabolic pathways.

Importantly, postmortem microbiota changes are organ specific [37]. Specifically, the microbial composition in postmortem organs (Fig. 1) differed. *Acinetobacter*, *Cupriavidus*, and *Ochrobactrum*, dominant in postmortem brain samples, are environmental organisms, especially in soil, and are considered opportunistic pathogens in humans in the hospital. *Enhydrobacter* is reportedly a member of the mammalian intestinal microbiome and was dominant in postmortem kidney and liver samples. The possible reason may be that the locations of the liver and kidney are near the intestine of the body. *Thermus*, dominant in the heart, liver, and kidney samples, was found in the human corpses [38]. Based on the comparison of alpha diversity, a lower observed ASV value was observed in the brain than in the other organs (*P* < 0.05, Fig. 4), which suggested that postmortem microbial diversity depends on the distance between the organ and the gut. The kidney and liver, located close to the gut, begin to putrefy in a specific natural order at the early stage of decomposition [37]. PCoA based on the beta diversity index showed a tendency to separate all the organ groups at the very beginning of decomposition, while the tendency was not clear over time (Fig. 4). Likewise, LEfSe analysis demonstrated that no difference remained after 1 day of decomposition (Fig. 5). All of the results confirmed that the compositions of the microbes participating in organ degradation became much more similar over time [39]. To further detected significant microbes of different organs, the continuous significant genera in different organs based on the *P*-value and LDA score of LEfSe analysis were summarized (Table 1). *Ochrobactrum*, *Agrobacterium*, *Leptothrix*, *Aminobacter*, *Bradyrhizobium*, and *Phyllobacterium* had significantly higher relative abundances in postmortem brain samples than in other organs during 1 day of decomposition. *Ochrobactrum* [40] and *Agrobacterium* [41] were reported to be associated with corpse decomposition. *Leptothrix* can be found in environments with sufficient amounts of organic matter, which may suggest its decomposition capacity. *Bradyrhizobium* has been reported to have the capacity to oxidize sulfur [42], carry out denitrification, and fix nitrogen. *Phyllobacterium* was reported to exhibit obvious changes in the postmortem oral microbiota community [43], which may suggest that it invades the brain from the mouth after death. *Sphingomonas* was found to be a continuous significant genus of heart compared to other organs and was also reported in internal organs of human corpses [37]. This genus is considered to comprise ammonia-oxidizing bacteria that can decompose polycyclic aromatic hydrocarbons positive for nitrogenase [44]. In addition, it has been reported produces NH_3_ using pig carcass proteins as a substrate [45]. *Lysinibacillus* and *Perlucidibaca* were identified as significant differential genera in the kidney compared to other organs, and these genera have been reported in other decomposition studies [46].

The differences among organs lie not only in the microbial composition but also in the relative abundance of some metabolic pathways. Selenium is widely distributed throughout the body, especially in the brain, even under dietary selenium deficiency [47]. When the body dies, selenocompounds in the brain degrade, as the relative abundance of selenocompound metabolism was higher in the H0.5Brain group than in the other organ groups [48]. The histidine metabolism pathway was activated in the H0.5Brain group but not in other organs. Histamine in the brain is a neurotransmitter formed by the decarboxylation of histidine and regulates diverse physiological functions. Histamine N-methyltransferase (HNMT) is a histamine-metabolizing enzyme expressed in the brain. These results all explained the differential abundance of pathways among different organs [49]. Several amino acid metabolism pathways, including those for D-alanine, glycine, serine, and threonine metabolism, were activated in the brain compared to those of the other organs, possibly because the protein content is highest in the brain. In the H0.5Kidney samples, the relative abundances of the genes associated with the bacterial chemotaxis and flagellar assembly pathways were lower than those in the other organs. This result may imply that the chemicals such as nutrients and toxins that drive microbial movement were not released yet [48, 50]. After 4 h of decomposition, the relative abundances of the pantothenate and CoA biosynthesis, selenocompound metabolism, and amino acid metabolism pathways were higher in the H4Brain group than in the other organ groups. In some bacteria, the production of phosphopantothenate by pantothenate kinase is the rate-limiting and most regulated step in the biosynthetic pathway [51]. Similar to those in the H0.5Brain group, some amino acid metabolism pathways, such as those for glycine, serine, threonine, histidine, alanine, aspartate, glutamate, and lysine, were activated because of some bacterial behavior. For example, the lysine biosynthetic pathway is closely associated with bacterial activity. Two products of this pathway, lysine, and mesodiaminopimelate (m-DAP), are directly involved in bacterial cell wall synthesis [52]. After 8 h of decomposition, biotin, lipoic acid metabolism, carbon fixation pathways in prokaryotes, and the terpenoid backbone biosynthesis pathway were inhibited in brain samples compared to the other organ samples. Biotin is a water-soluble vitamin that functions as a cofactor of enzymes involved in carboxylation reactions to maintain metabolic homeostasis [53]. Lipoic acid, an enzyme cofactor, is related to lipoylation strategies of microbial pathogens, which can affect pathogenesis and virulence [54]. The lipoic acid metabolism, relatively activated in the heart, liver, and kidney, may suggest the proliferation of pathogens in these organs. After 12 h of decomposition, the pyruvate metabolism pathway was enriched in the kidney compared with the other organs. Lactic acid bacteria can produce pyruvate and lactate by the use of carbohydrates, organic acids, and amino acids. The LEfSe analysis results also revealed *Lactobacillales* as a significant bacterium in H12Kidney samples in contrast to in the other organs at 12 h. This study described and compared the microbial community composition among different organs in a 1-day decomposing cadaver in detail. The functional prediction of the microbial community in the early decomposition body was also analyzed in this work. The knowledge of microbial composition in H0.5 groups would facilitate organ transplantation because some donor organs come from deceased individuals and the microbes may affect the acceptance of the organ. The microbial markers from the thanatomicrobiome would be helpful in the risk reduction of de novo malignancy post-organ transplantation. Antagonizing the microbes of donor organs could reduce the failure rate of organ transplants in recipients [16, 55]. *Lactobacillus* has been reported the highest detection rate in organs at 3 and 12 h postmortem [56]. Similarly, the relative abundance of this bacteria increased during 12 h of decomposition, especially in postmortem kidney samples. The rapid translocation of intestinal bacteria could be beneficial for screening organ donors before allogeneic transplantation [56].

However, there are still some limitations to this study. To give a basic look at the microbial community succession of internal organs during decomposition, a controlled-environment chamber was used for place remains. Some factors interfered with body decomposition in a real case, such as humidity, temperature, and insect behavior. Based on the results of the current study, the influence of the outside environment and complex inter-reaction with the hosts should be studied in the future. Besides, the sample size of this study was limited, which may make the results of the statistical analysis less conclusive. Future large-scale studies investigating human remains decomposition in mortality events will expand the results of this work.

## Conclusion

In conclusion, postmortem internal organs contained bacteria from 0.5 h to 24 h after death but had a relatively low species richness and abundance of bacteria. Interestingly, the microbiome changed after death, and more importantly, these alterations were organ-specific. The dominant microbes differed from one organ to another, while they tended to become more similar over time. Substantial differences in the microbial relative change rate between different organs over time due to the initial microbial composition differed. The thanatomicrobiome variation by body site provides new knowledge into decomposition ecology. These findings may also be valuable in early PMI estimation and organ transplantation.

## Methods

### Sample collection protocol

Adult male C57BL/6 J mice (SPF, 18–25 g, 6–10 weeks, *n* = 30) were acquired from the Experimental Animal Center of Xi’an Jiaotong University and cohoused under standard lighting (light/dark periods of 12 h), temperature (25.0 ± 1.5 °C), and relative humidity (50.0 ± 7.0%) conditions at the animal centre of Xi’an Jiaotong University. They were housed and handled daily under laboratory conditions for at least 7 days before the start of the study. Water and food were available ad libitum. All animal experiments were approved by the Committee of the Ethics of Animal Experiments of Xi’an Jiaotong University (IRB: 2017–388). The experiment was performed under the same conditions of light, temperature, and relative humidity described above. Mice were euthanized by isoflurane inhalation followed by cervical dislocation. After cervical dislocation, mice were placed in a controlled-environment chamber (PRX-350D, ZuoLe, ShangHai) with constant temperature (25.0 ± 1.5 ℃) and relative humidity (50.0 ± 7.0%) at Xi’an Jiaotong University, College of Forensic Medicine. Internal organic samples (brain, heart, liver, and kidneys) were harvested from mouse corpses following aseptic principles at half an hour, 4 h, 8 h, 12 h, and 1 day after death (6 mice per timepoint at random). Concretely, every surgical instrument was autoclaved before being used. The clean bench was sterilized under ultraviolet light for at least 2 h before the operation. Operators wore sterile surgical clothes during the operation. In addition, surgical instruments were swabbed before dissection with sterile swabs and set as negative controls. 120 samples were then immediately stored at − 80 °C until further experimentation.

### Postmortem microbiota analysis by 16S rRNA gene sequencing

Total genomic DNA was extracted from samples by OMEGA Soil DNA Kit (D5625-01) (Omega Bio-Tek, Norcross, GA, USA) according to the manufacturer’s recommendations. The quantity and quality of extracted DNA were evaluated by NanoDrop ND-1000 spectrophotometer (Thermo Fisher Scientific, Waltham, MA, USA) and agarose gel electrophoresis. The microbial 16S rRNA gene V3 and V4 regions were amplified by polymerase chain reaction (PCR) with the universal primers 338F (5'-ACTCCTACGGGAGCAGCAGCA-3') and 806R (5'-GGACTACVSGTATCTAAT-3'). The PCR components consisted of 5 μl of buffer (5 ×), 0.25 μl of DNA Polymerase (5 U/μl), 2 μl (2.5 mM) of dNTPs, 1 μl (10 µM) of 338F and 806R primer, 1 μl of DNA template, and 14.75 μl of ddH_2_O. The PCR cycling included an initial denaturation for 30 s at 98 °C, 30 cycles annealing of 15 s at 98 °C, 30 s at 50 °C, and 30 s at 72 °C, and an extension for 5 min at 72 °C. PCR products were purified with Vazyme VAHTSTM DNA Clean Beads (Vazyme, Nanjing, China). quantified by a Quant-iT PicoGreen dsDNA Assay Kit (Invitrogen, Carlsbad, CA, USA). Equal amount of amplicons were paired-end 2 × 250 bp sequenced on the Illumina MiSeq platform with a MiSeq Reagent Kit v3.

### Sequence bioinformatics analysis

Microbiome bioinformatic analysis was performed on the QIIME2 platform [57] following the official tutorials (https://docs.qiime2.org/2019.4/tutorials/), with slight modification. In brief, we demultiplex the raw sequence data using the demux plugin followed by primer trimming with the cutadapt plugin [58]. The sequence data were then quality filtered, denoised, merged, and cleaned from chimeric reads using the DADA2 plugin [59]. Nonsingleton amplicon sequence variants (ASVs) were aligned with mafft [60] and used to construct a phylogeny with fasttree2 [61]. The richness and evenness of ASVs among samples were compared by generating ASV-level ranked abundance curves. The classify-sklearn naïve Bayes taxonomy classifier [62] plugin with the SILVA Release 132 Database was used to assign taxonomy to the ASVs. 16S rRNA gene sequence data analyses were mainly performed with QIIME2 and R packages (v3.2.0). Alpha diversity metrics included Chao1 [63], observed species, Shannon [64], Simpson [65], Faith’s phylogenetic diversity (PD) [66], Pielou’s evenness [67] and Good’s coverage [68]. Beta diversity metrics included Jaccard metrics, Bray–Curtis metrics, and UniFrac distance [69, 70]. The PCoA, nonmetric multidimensional scaling (NMDS), and unweighted paired-group method with arithmetic means (UPGMA) hierarchical clustering were utilized to visualize the structural variation in microbial communities [71]. Permutational multivariate analysis of variance (PERMANOVA) was applied to assess the significance of microbial community structure differentiation among groups [72]. The taxonomy compositions and abundances were visualized using MEGAN [73] and GraPhlAn [74]. LEfSe analysis was utilized to detect statistically significant differences between groups. The LDA > 3 and *P* < 0.05 was were considered significantly enriched in that group compared to other groups. PICRUSt2 was applied to predict functional information [25] with the KEGG (https://www.kegg.jp/) databases. Network analysis based on Spearman's rank correlation coefficient (r) was conducted using taxa at the genus level. The co-occurrence network patterns of microbial communities were further examined for OTUs with strong (*r* > 0.6, *r* <  − 0.6) and significant (*P* < 0.01) correlations. The networks were visualized using Cytoscape [75].

### Statistical analysis

The statistical analyses in this work were conducted using SPSS software (v18.0, Chicago, IL, USA). Statistical analyses used in this study were Student’s t-test, a least significant difference (LSD), Kruskal–Wallis rank-sum test (KW-test), analysis of variance (ANOVA), and Dunnett’s T3 test according to the applicable conditions. The student’s t-test generated a *P* value. The * represents *P* < 0.05 (confidence interval of 95%), the ** represents *P* < 0.1 (confidence interval of 90%), and the *** represents *P* < 0.001 (confidence interval of 99.9%). Statistical tests, sample size, and exact *P* value are indicated in relevant figure legends.

## Supplementary Information

**Additional file 1: Fig. S1.** Taxonomic profiles of postmortem microbial communities in internal organs during decomposition (community composition was based on the top 20 genera). (A-D): Plots are representative of the brain, heart, liver, and kidney groups, respectively. The other levels, such as phyla, orders, and species, are illustrated in the supplementary materials (Fig. S2). **Fig. S2.** Taxonomic profiles of postmortem microbial communities in internal organs during decomposition (community composition was based on the top 20 phyla, orders, and species). (A-D): Plots are representative of the brain, heart, liver, and kidney groups, respectively. **Fig. S3.** Alpha diversity within subjects at different PMIs in different internal organs as measured using Chao1, observed species, Faith‘s PD, Pielou’s evenness, and Good’s coverage. (A-C): Plots demonstrated the Pielou’s evenness, Faith‘s PD, and Good’s coverage index comparisons between brain groups. (D) demonstrated Faith‘s PD index comparison between liver groups. (E) demonstrated observed species index comparison between kidney groups. (F) demonstrated Chao1 index comparison between kidney groups. *P value< 0.05, **P value < 0.01, ***P value< 0.001. **Fig. S4.** Network diagram of genera correlation in brain samples. (A) H0.5Brain, (B) H4Brain, (C) H8Brain, (D) H12Brain, and (E) H24Brain. Nodes with different colors represented different genera; node size represented genera abundances; the color of the line represented correlations (red and green lines represented positive and negative correlations, respectively). **Fig. S5.** Network diagram of genera correlation in heart samples. (A) H0.5Heart, (B) H4Heart, (C) H8Heart, (D) H12Heart, and (E) H24Heart. Nodes with different colors represented different genera; node size represented genera abundances; the color of the line represented correlations (red and green lines represented positive and negative correlations, respectively). **Fig. S6.** Network diagram of genera correlation in liver samples. (A) H0.5Liver, (B) H4Liver, (C) H8Liver, (D) H12Liver, and (E) H24Liver. Nodes with different colors represented different genera; node size represented genera abundances; the color of the line represented correlations (red and green lines represented positive and negative correlations, respectively). **Fig. S7.** Network diagram of genera correlation in kidney samples. (A) H0.5Kidney, (B) H4Kidney, (C) H8Kidney, (D) H12Kidney, and (E) H24Kidney. Nodes with different colors represented different genera; node size represented genera abundances; the color of the line represented correlations (red and green lines represented positive and negative correlations, respectively). **Fig. S8.** LEfSe analysis of the metabolic pathway in brain samples. (A) H0.5Brain v.s. H8Brain, (B) H0.5Brain v.s. H12Brain, (C) H0.5Brain v.s. H24Brain, (D) H24Brain v.s. H4Brain, (E) H4Brain v.s. H8Brain, (F) H12Brain v.s. H8Brain. Different colors were regarded as different *P* value, green represented *P* < 0.05; orange represented *P* < 0.01; red represented *P* < 0.001. When group A v.s. group B, the positive value of logFC meant that the relative abundance of this pathway was higher in group B than in group A. **Fig.S9.** LEfSe analysis of the metabolic pathway in heart, liver, and kidney samples. (A) H4Heart v.s. H8Heart, (B) H0.5Liver v.s. H12Liver, (C) H12Liver v.s. H4Liver, (D) H0.5Liver v.s. H24Liver, (E) H12Liver v.s. H24Liver, (F) H12Liver v.s. H8Liver, (G) H24Kidney v.s. H4Kidney, (H) H24Kidney v.s. H8Kidney. Different colors were regarded as different *P* value, green represented *P* < 0.05; orange represented *P* < 0.01; red represented *P* < 0.001. When group A v.s. group B, the positive value of logFC meant that the relative abundance of this pathway was higher in group B than in group A.

## Data Availability

All raw sequence data used for our analyses have been uploaded to the National Center for Biotechnology Information Sequence Read Archive (NCBI-SRA) under BioProject number PRJNA746418: www.ncbi.nlm.nih.gov/bioproject/PRJNA746418. Four supplementary figures are available with the online version of this article.

## Abbreviations

PMI: Postmortem interval
PCoA: Principal coordinate analysis
PERMANOVA: Permutational analyses of variance
16S rRNA: 16S ribosomal RNA
EPMI: Early postmortem interval (within 1 day)
LPMI: Long postmortem interval (greater than 1 day)
PCR: Polymerase chain reaction
ASVs: Nonsingleton amplicon sequence variants
Faith’s PD: Faith’s phylogenetic diversity
NMDS: Non-metric multidimensional scaling
UPGMA: Unweighted paired-group method with arithmetic means
LEfSe: Linear discriminant analysis effect size
PICRUSt2: Phylogenetic investigation of communities by reconstruction of unobserved states 2
KEGG: Kyoto Encyclopedia of Genes and Genomes
LSD: Least significant difference
KW-test: Kruskal–Wallis rank-sum test
ANOVA: Analysis of variance
FISH: Fluorescence in situ hybridization
