# Supplementary material for: Dissecting the microbial community structure of internal organs during the early postmortem period in a murine corpse model
Source: BMC Microbiol. 2023 Feb 10;23:38. doi: 10.1186/s12866-023-02786-0 (PMC9912631; doi:10.1186/s12866-023-02786-0)
Supplement: Supplementary file 1 — Additional file 1: Fig. S1. Taxonomic profiles of postmortem microbial communities in internal organs during decomposition (community composition was based on the top 20 genera). (A-D): Plots are representative of the brain, heart, liver, and kidney groups, respectively. The other levels, such as phyla, orders, and species, are illustrated in the supplementary materials (Fig. S2). Fig. S2. Taxonomic profiles of postmortem microbial communities in internal organs during decomposition (community composition was based on the top 20 phyla, orders, and species). (A-D): Plots are representative of the brain, heart, liver, and kidney groups, respectively. Fig. S3. Alpha diversity within subjects at different PMIs in different internal organs as measured using Chao1, observed species, Faith‘s PD, Pielou’s evenness, and Good’s coverage. (A-C): Plots demonstrated the Pielou’s evenness, Faith‘s PD, and Good’s coverage index comparisons between brain groups. (D) demonstrated Faith‘s PD index comparison between liver groups. (E) demonstrated observed species index comparison between kidney groups. (F) demonstrated Chao1 index comparison between kidney groups. *P value< 0.05, **P value < 0.01, ***P value< 0.001. Fig. S4. Network diagram of genera correlation in brain samples. (A) H0.5Brain, (B) H4Brain, (C) H8Brain, (D) H12Brain, and (E) H24Brain. Nodes with different colors represented different genera; node size represented genera abundances; the color of the line represented correlations (red and green lines represented positive and negative correlations, respectively). Fig. S5. Network diagram of genera correlation in heart samples. (A) H0.5Heart, (B) H4Heart, (C) H8Heart, (D) H12Heart, and (E) H24Heart. Nodes with different colors represented different genera; node size represented genera abundances; the color of the line represented correlations (red and green lines represented positive and negative correlations, respectively). Fig. S6. Network diagram of genera correlation [file 12866_2023_2786_MOESM1_ESM.docx]

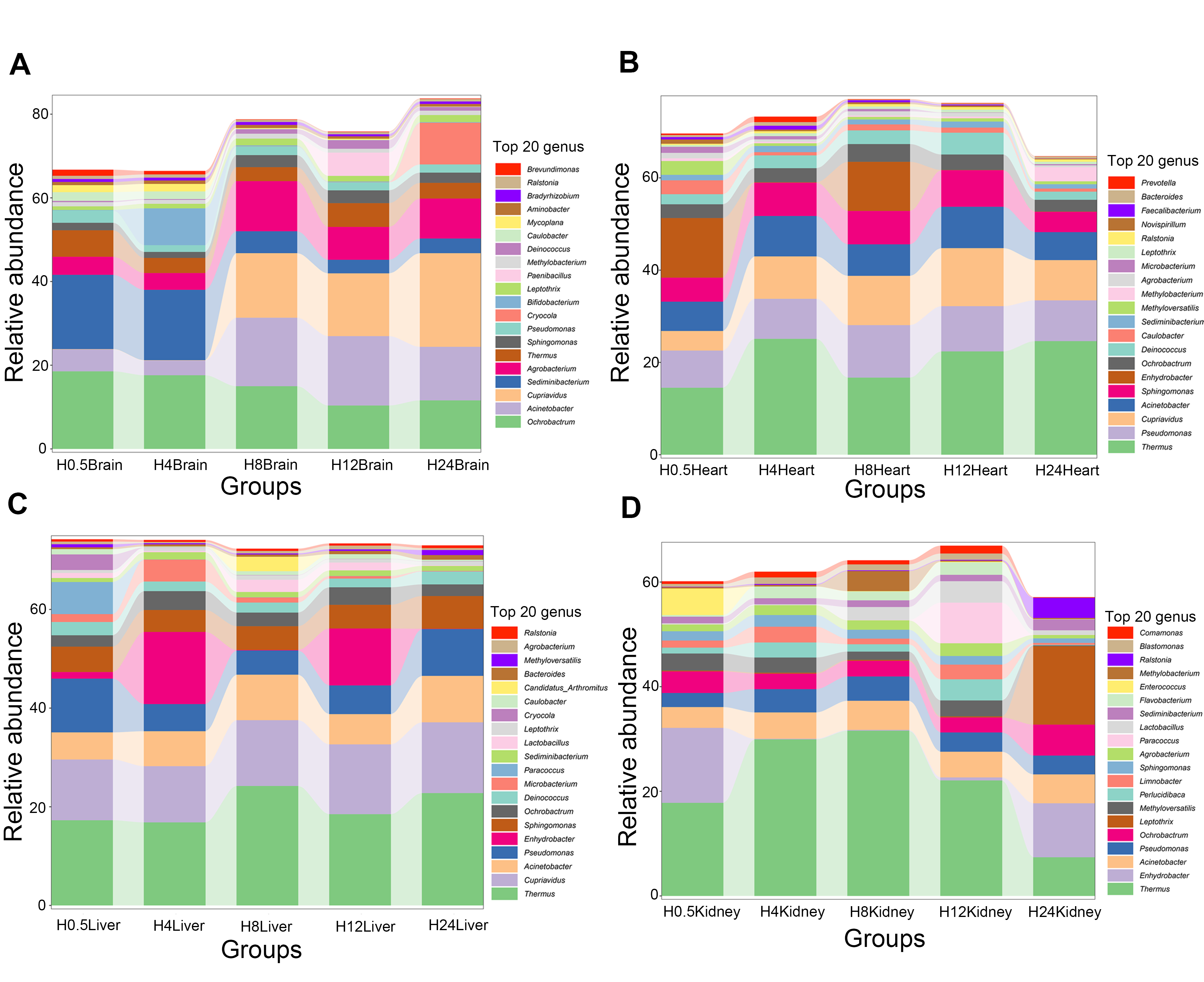
Fig. S1 Taxonomic profiles of postmortem microbial communities in internal organs during decomposition (community composition was based on the top 20 genera). (A-D): Plots are representative of the brain, heart, liver, and kidney groups, respectively. The other levels, such as phyla, orders, and species, are illustrated in the supplementary materials (Fig. S2).


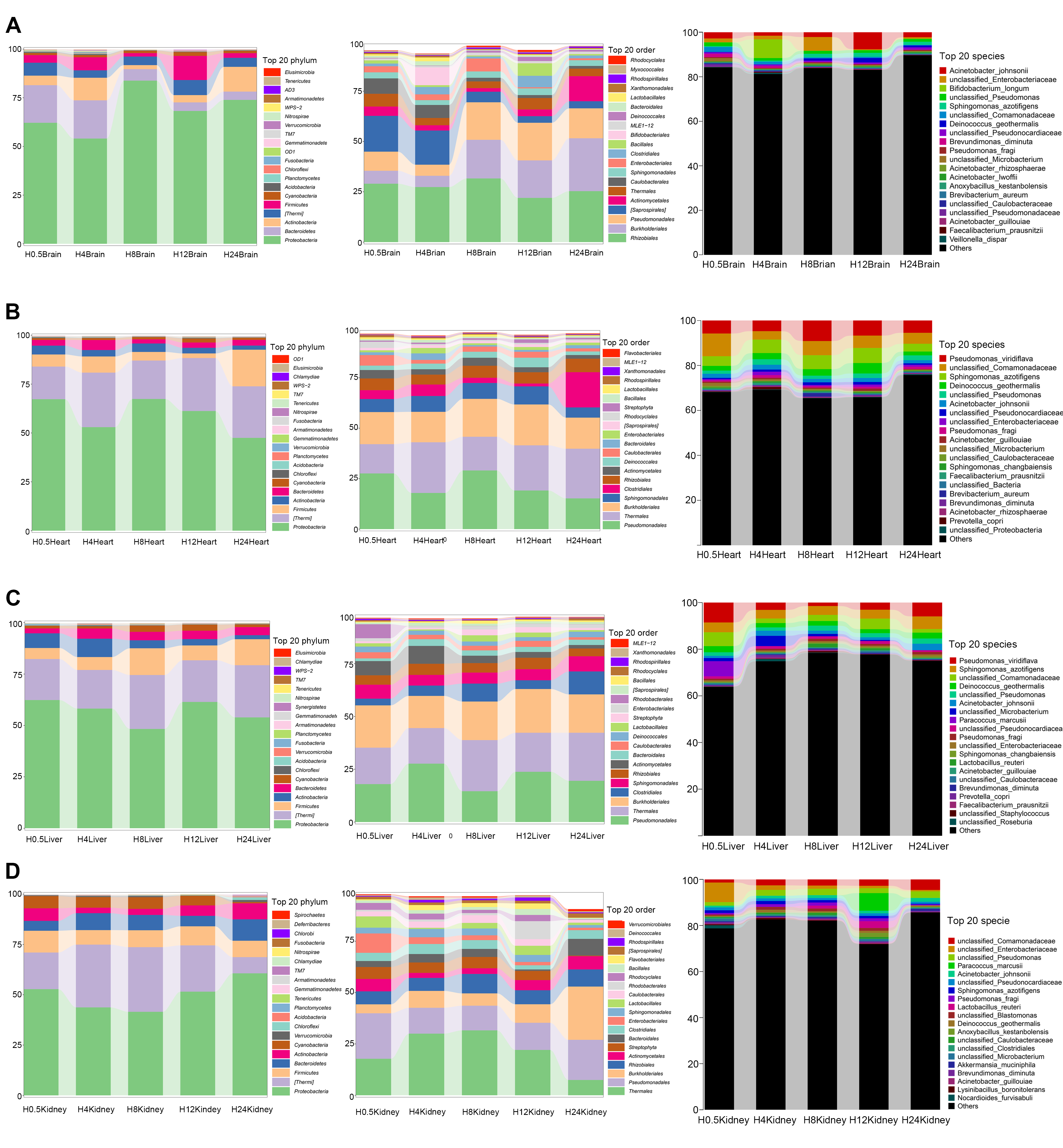


Fig. S2 Taxonomic profiles of postmortem microbial communities in internal organs during decomposition (community composition was based on the top 20 phyla, orders, and species). (A-D): Plots are representative of the brain, heart, liver, and kidney groups, respectively.


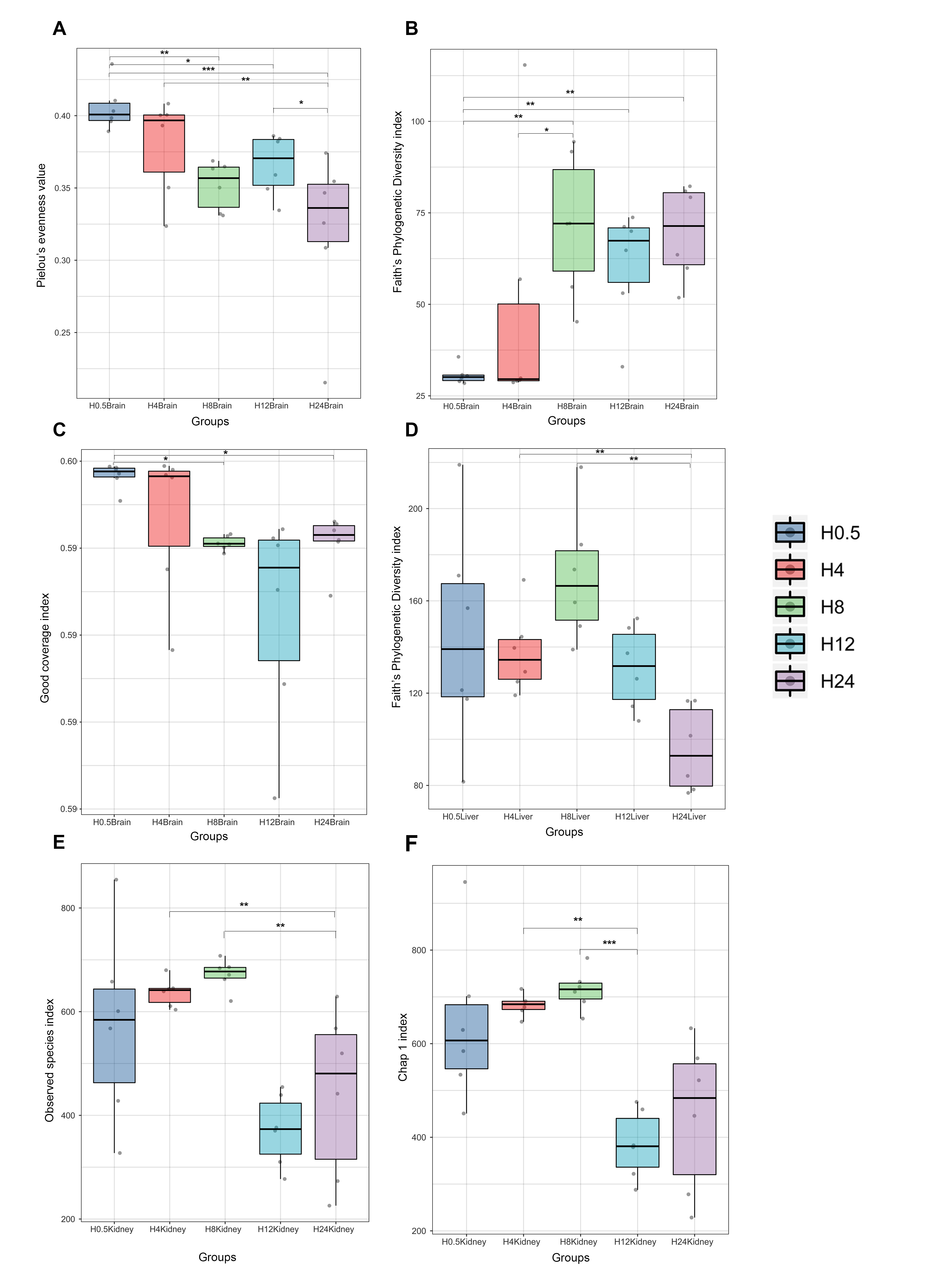


Fig. S3 Alpha diversity within subjects at different PMIs in different internal organs as measured using Chao1, observed species, Faith‘s PD, Pielou’s evenness, and Good’s coverage. (A-C): Plots demonstrated the Pielou’s evenness, Faith‘s PD, and Good’s coverage index comparisons between brain groups. (D) demonstrated Faith‘s PD index comparison between liver groups. (E) demonstrated observed species index comparison between kidney groups. (F) demonstrated Chao1 index comparison between kidney groups. **P* value< 0.05, ***P* value < 0.01, ****P* value< 0.001.


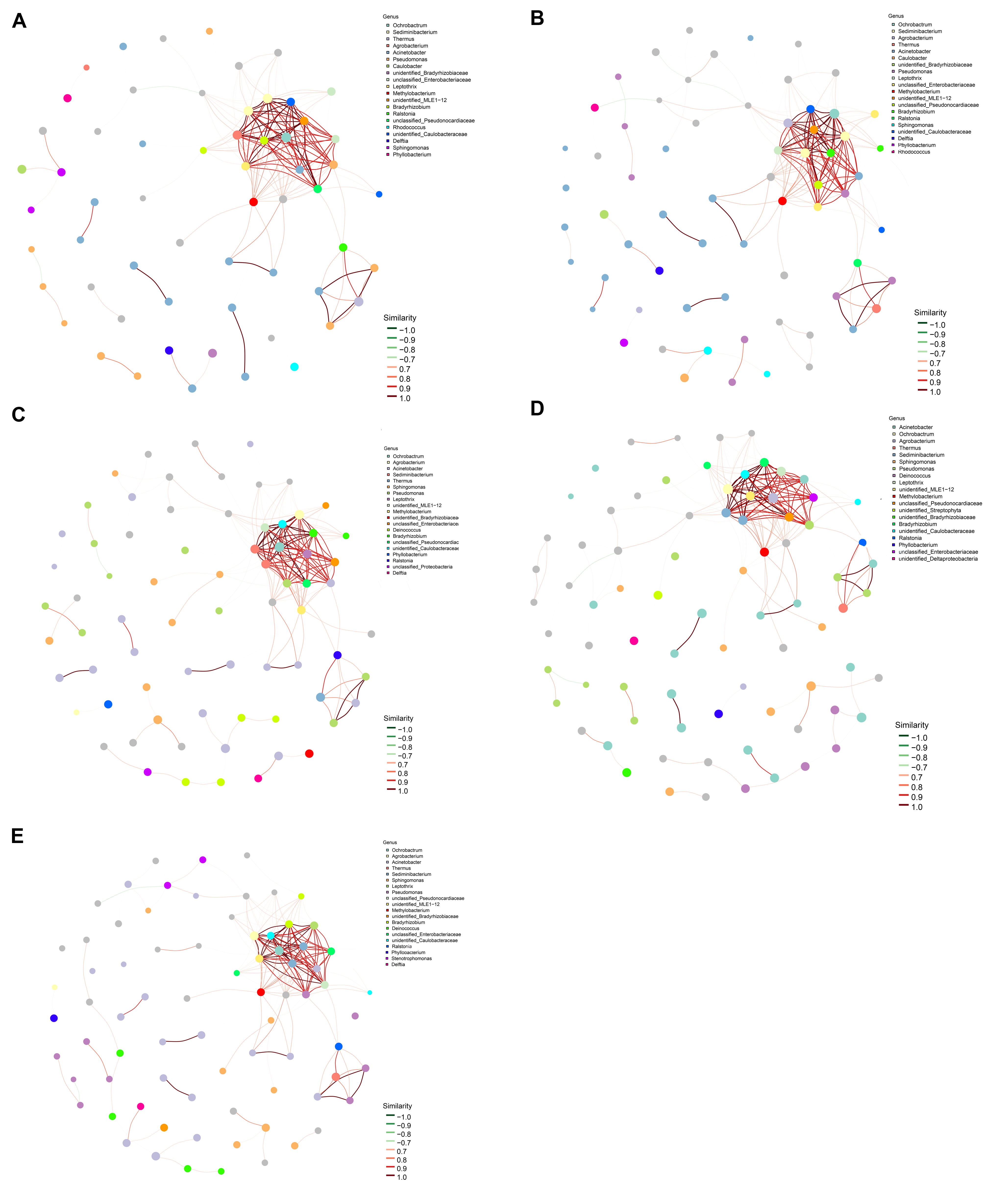


Fig. S4 Network diagram of genera correlation in brain samples. (A) H0.5Brain, (B) H4Brain, (C) H8Brain, (D) H12Brain, and (E) H24Brain. Nodes with different colors represented different genera; node size represented genera abundances; the color of the line represented correlations (red and green lines represented positive and negative correlations, respectively).


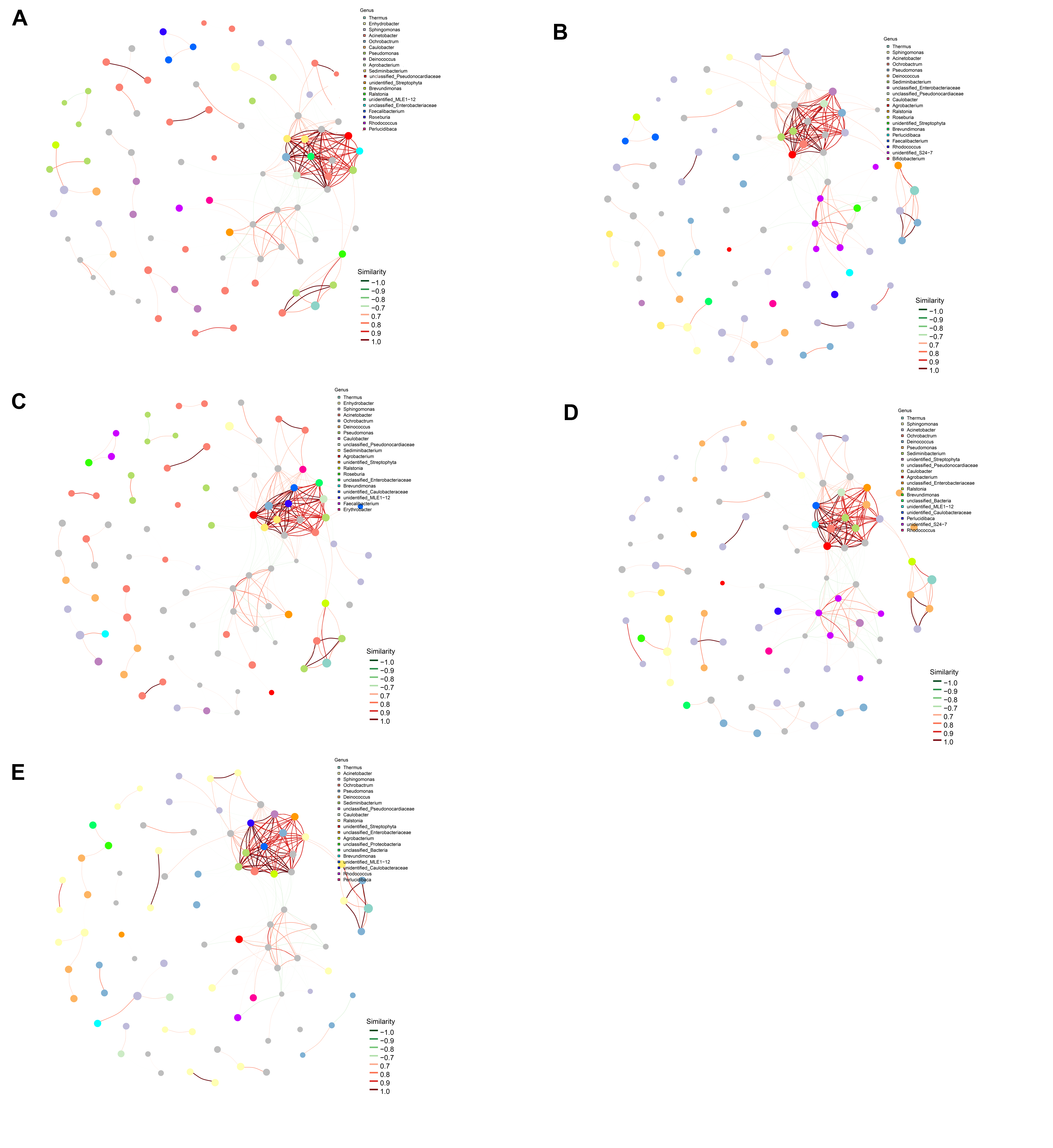


Fig. S5 Network diagram of genera correlation in heart samples. (A) H0.5Heart, (B) H4Heart, (C) H8Heart, (D) H12Heart, and (E) H24Heart. Nodes with different colors represented different genera; node size represented genera abundances; the color of the line represented correlations (red and green lines represented positive and negative correlations, respectively).


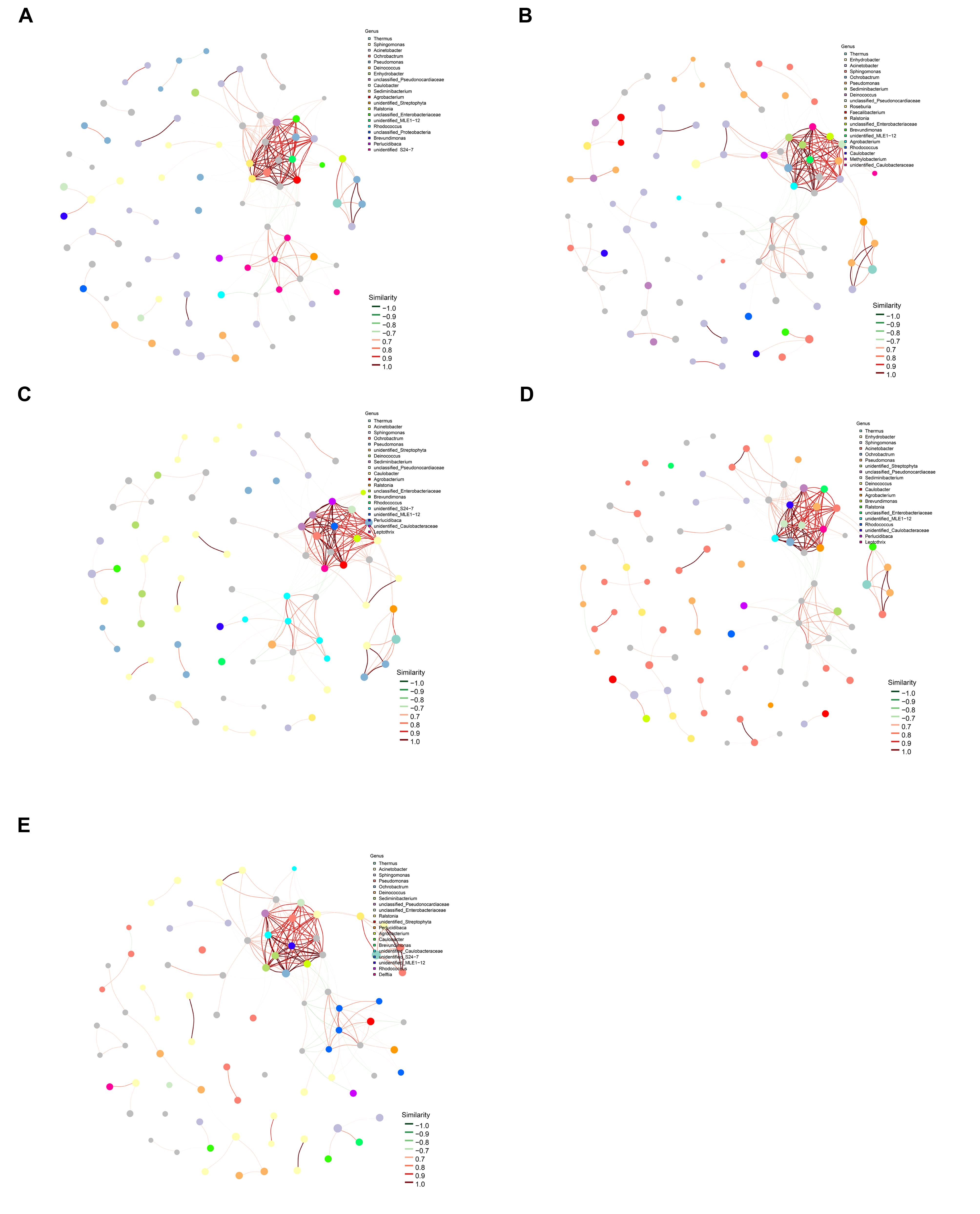


Fig. S6 Network diagram of genera correlation in liver samples. (A) H0.5Liver, (B) H4Liver, (C) H8Liver, (D) H12Liver, and (E) H24Liver. Nodes with different colors represented different genera; node size represented genera abundances; the color of the line represented correlations (red and green lines represented positive and negative correlations, respectively).


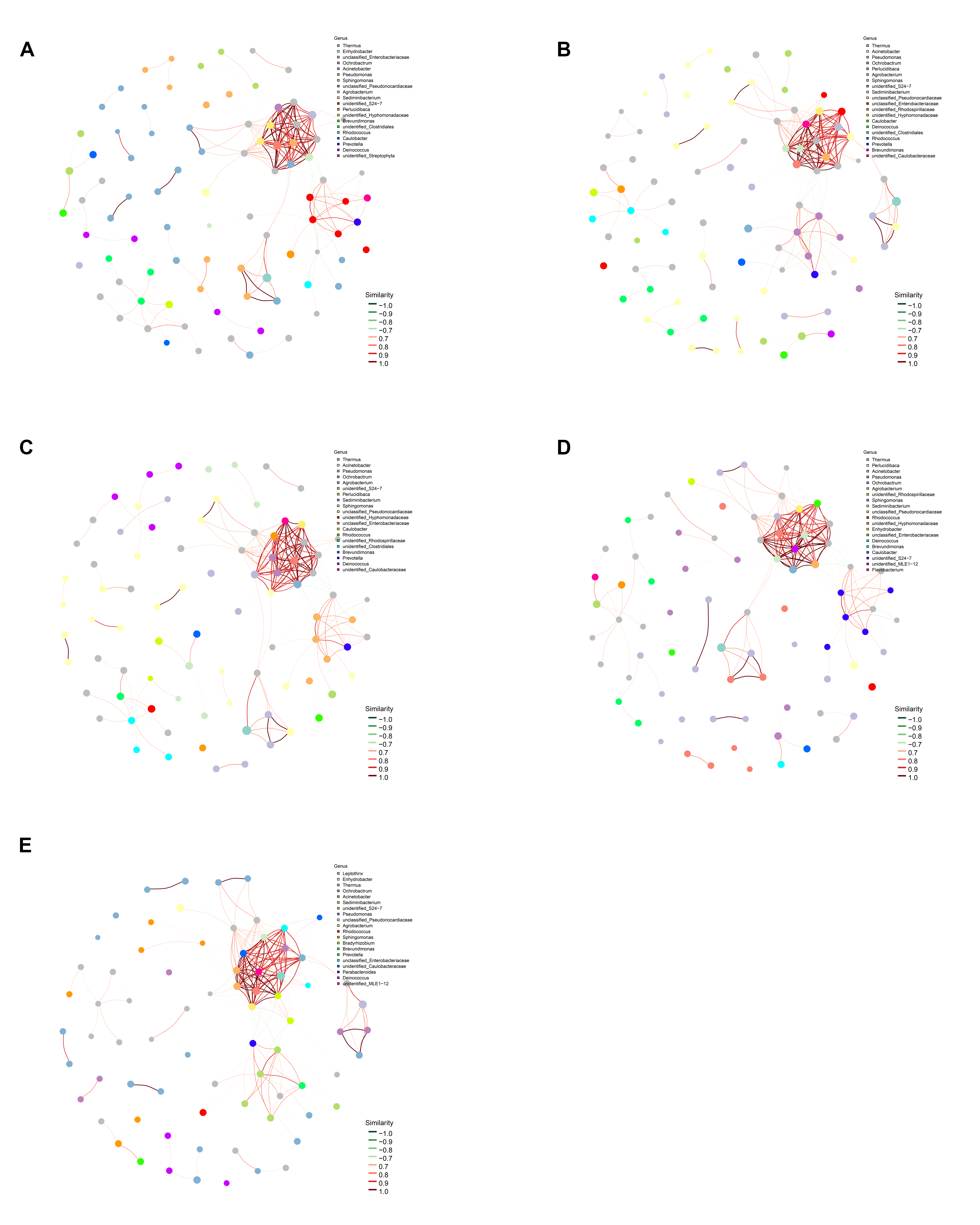


Fig. S7 Network diagram of genera correlation in kidney samples. (A) H0.5Kidney, (B) H4Kidney, (C) H8Kidney, (D) H12Kidney, and (E) H24Kidney. Nodes with different colors represented different genera; node size represented genera abundances; the color of the line represented correlations (red and green lines represented positive and negative correlations, respectively).


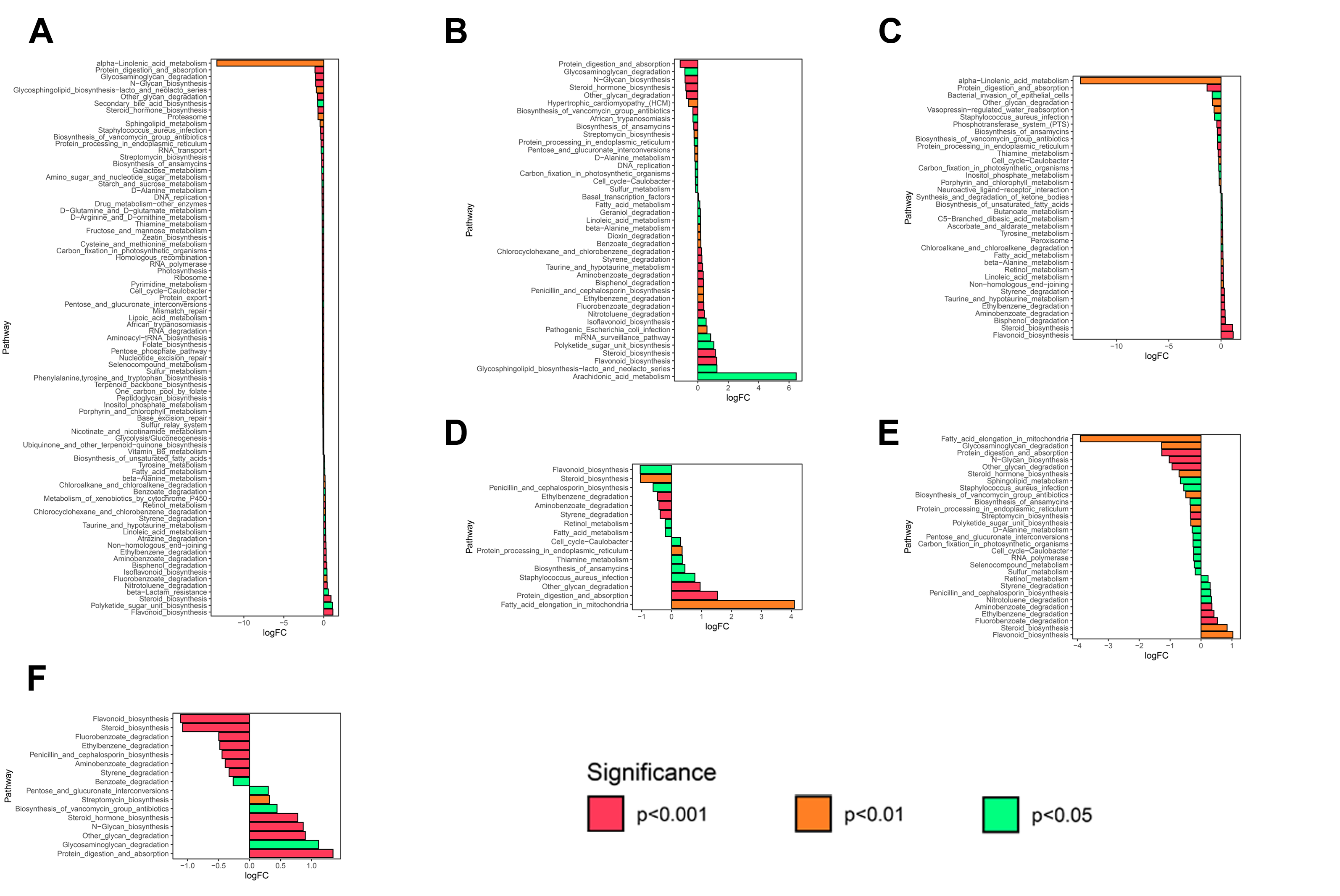


Fig. S8 LEfSe analysis of the metabolic pathway in brain samples. (A) H0.5Brain v.s. H8Brain, (B) H0.5Brain v.s. H12Brain, (C) H0.5Brain v.s. H24Brain, (D) H24Brain v.s. H4Brain, (E) H4Brain v.s. H8Brain, (F) H12Brain v.s. H8Brain. Different colors were regarded as different *P* value, green represented *P* < 0.05; orange represented *P* < 0.01; red represented *P* < 0.001. When group A v.s. group B, the positive value of logFC meant that the relative abundance of this pathway was higher in group B than in group A.


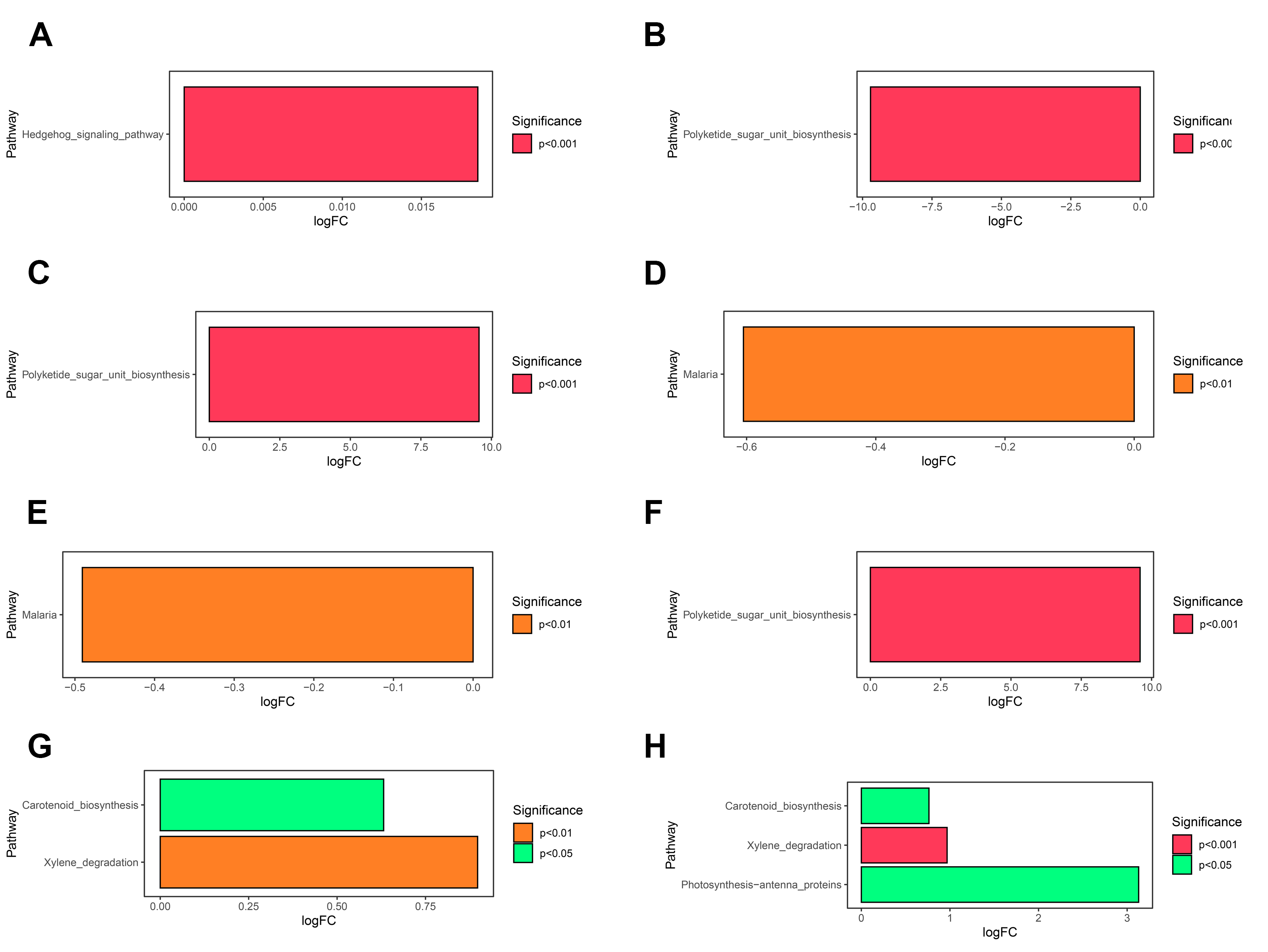


Fig.S9 LEfSe analysis of the metabolic pathway in heart, liver, and kidney samples. (A) H4Heart v.s. H8Heart, (B) H0.5Liver v.s. H12Liver, (C) H12Liver v.s. H4Liver, (D) H0.5Liver v.s. H24Liver, (E) H12Liver v.s. H24Liver, (F) H12Liver v.s. H8Liver, (G) H24Kidney v.s. H4Kidney, (H) H24Kidney v.s. H8Kidney. Different colors were regarded as different *P* value, green represented *P* < 0.05; orange represented *P* < 0.01; red represented *P* < 0.001. When group A v.s. group B, the positive value of logFC meant that the relative abundance of this pathway was higher in group B than in group A.
